# Human 4E-T represses translation of bound mRNAs and enhances microRNA-mediated silencing

**DOI:** 10.1093/nar/gkt1265

**Published:** 2013-12-13

**Authors:** Anastasiia Kamenska, Wei-Ting Lu, Dorota Kubacka, Helen Broomhead, Nicola Minshall, Martin Bushell, Nancy Standart

**Affiliations:** ^1^Department of Biochemistry, University of Cambridge, Tennis Court Road, Cambridge CB21QW, UK and ^2^MRC Toxicology Unit, University of Leicester, Lancaster Road, Leicester LE19HN, UK

## Abstract

A key player in translation initiation is eIF4E, the mRNA 5′ cap-binding protein. 4E-Transporter (4E-T) is a recently characterized eIF4E-binding protein, which regulates specific mRNAs in several developmental model systems. Here, we first investigated the role of its enrichment in P-bodies and eIF4E-binding in translational regulation in mammalian cells. Identification of the conserved C-terminal sequences that target 4E-T to P-bodies was enabled by comparison of vertebrate proteins with homologues in *Drosophila* (Cup and CG32016) and *Caenorhabditis elegans* by sequence and cellular distribution. In tether function assays, 4E-T represses bound mRNA translation, in a manner independent of these localization sequences, or of endogenous P-bodies. Quantitative polymerase chain reaction and northern blot analysis verified that bound mRNA remained intact and polyadenylated. Ectopic 4E-T reduces translation globally in a manner dependent on eIF4E binding its consensus Y^30^X_4_Lϕ site. In contrast, tethered 4E-T continued to repress translation when eIF4E-binding was prevented by mutagenesis of YX_4_Lϕ, and modestly enhanced the decay of bound mRNA, compared with wild-type 4E-T, mediated by increased binding of CNOT1/7 deadenylase subunits. As depleting 4E-T from HeLa cells increased steady-state translation, in part due to relief of microRNA-mediated silencing, this work demonstrates the conserved yet unconventional mechanism of 4E-T silencing of particular subsets of mRNAs.

## INTRODUCTION

Regulation of translation is critical in the control of cell growth and proliferation. A key player in translation initiation is eIF4E, the mRNA 5′ cap-binding protein. Aberrant expression of eIF4E and its phosphorylation promotes tumorigenesis and has been implicated in cancer development. eIF4E recruits ribosomes to mRNA 5′ ends through specific and high-affinity binding to eIF4G. Translation initiation requires the rate-limiting binding of eIF4F (eIF4E, eIF4G and the RNA helicase eIF4A) to the m^7^G cap structure and is completed on initiation codon recognition by the preinitiation complex. eIF4E sandwiches the cap via conserved tryptophan residues, and its convex side interacts with the YXXXXLϕ sequence in eIF4G. General inhibitors of translation initiation known as eIF4E-binding proteins (4E-BPs) contain similar YX_4_Lϕ motifs, and thus can be considered as molecular mimics of eIF4G, which compete for the same binding site of eIF4E, blocking the formation of the translation initiation complex, when hypophosphorylated in quiescent cells (1–4).

Additional eIF4E-binding proteins have been reported that regulate specific mRNAs, the best characterized of these being the vertebrate 4E-Transporter (also known as eIF4NIF1) protein and its fly and worm homologues Cup and Spn-2 (Pqn-45 or IFET-1) that act in early development. In *Xenopus* oocytes, 4E-T is a component of the large 2–3 MDa CPEB RNP (ribonucleoprotein) translational repressor complex (5), which also contains Xp54 RNA helicase (rck/p54), and the RNA-binding proteins Pat1 and Rap55. Cytoplasmic polyadenylation element-binding protein (CPEB) binds and regulates the translation of maternal mRNA with 3′ UTR cytoplasmic polyadenylation elements, CPEs (6). Cup and Spn-2 repress translation of oskar, nanos and katanin mRNAs and their mutant alleles arrest oogenesis (7–15). Cup is enriched in nurse cell and oocyte RNP that contain oskar mRNA, Me31B (p54/rck) and eIF4E (9). Spn-2 localizes to the cytoplasm and P-granule RNP (10), ribonucleoprotein particles important for germ line development (16), and functions in conjunction with the broad-scale translational regulators CGH-1 (rck/p54), CAR-1 (Rap55) and PATR-1 (Pat1) (17). As with the much smaller 4E-BP proteins, binding of 4E-T and Cup to eIF4E prevents eIF4E-eIF4G interactions due to competition for the same binding site (18,19). Current models propose that 4E-T and homologues interact with 3′ UTR-RNA-binding proteins including CPEB, Bruno, Smaug and OMA-1/2 as well as with the cap-binding protein, but fail to recruit the small ribosomal subunit thus resulting in a repressive closed loop (3).

Human 4E-T (h4E-T) was first characterized as a nucleocytoplasmic shuttling protein with a canonical YX_4_Lϕ sequence that mediates eIF4E binding, and its nuclear export/import via Crm1 (18). With the exception of the eIF4E-binding site and the NLS/NES sequences, the primary structure of this 985 amino acid long protein gives no additional hints regarding function. While its nuclear role remains unclear, recent studies have focused on its cytoplasmic function with the discovery that h4E-T is enriched in distinct foci, P-(rocessing) bodies (20–22). Ectopic 4E-T was shown to inhibit cap-dependent reporter mRNA translation, but not IRES-mediated translation, in the absence of changes to reporter mRNA levels in HeLa cells (21,22). However, 4E-T RNAi stabilizes AU-rich (ARE)-reporter and -cellular mRNAs, though not globin mRNA (21,22), altogether suggesting that 4E-T regulates specific mRNAs, possibly those in P-bodies.

P-bodies, which contain mRNA, microRNAs, RNA-binding proteins/translational repressors and mRNA decay enzymes but not ribosomes, are thought to be involved in reversible translational repression including that mediated by microRNAs and in mRNA decay [reviewed by (23–25)]. To date, in mammalian tissue culture cells, eIF4E is the only translation initiation factor found enriched in P-bodies and 4E-T the only enriched eIF4E-binding protein. Strikingly, mRNP granules including P-bodies, maternal and neuronal mRNP granules, in organisms ranging from yeast, Plasmodium and trypanosomes to man, share overlapping, albeit not identical compositions, attesting to critical, conserved functions in gene regulation. Shared components of P-bodies/RNP granules include p54/rck, Rap55 and Pat1 (16,26).

The molecular details of the P-body pathway in gene expression control remain to be determined, and available evidence does not offer a simple functional conclusion. Individual components—for example, GW182 and rck/p54—have well-characterized important functions in general and in miRNP-mediated gene expression control. However, the visible presence of P-bodies is not required for reporter mRNA decay mediated by microRNAs, siRNAs or via the NMD pathway, nor for general mRNA decay/translational repression in yeast (27–29). On the other hand, ARE mRNAs have been shown to be degraded in mammalian P-bodies (30,31), and recent reports suggest that additional specific mRNA decay and/or translational repression may occur in these foci (32–35).

We were interested to investigate the role of P-bodies and eIF4E-binding in translational control mediated by 4E-T in mammalian cells, and to this end, we first delineated the sequences that target 4E-T to P-bodies. The deletion analysis of GFP-4E-T was based on comparison of sequences of vertebrate 4E-T proteins with their homologues in *Drosophila* (both Cup and the relatively uncharacterized CG32016 protein closer to human 4E-T than Cup) and *Caenorhabditis elegans*, and showed that conserved C-terminal sequences targeted 4E-T to P-bodies. In tether function assays, we showed that 4E-T represses translation of reporter mRNA in a P-body- and eIF4E-independent manner, and does not promote deadenylation. 4E-T mutated in its eIF4E-binding site modestly enhanced the decay of tethered mRNA, compared with wild-type 4E-T, due to increased binding of CNOT1/7 deadenylase subunits, likely accompanied by decapping. We also addressed the question of the cellular targets of 4E-T, and provide evidence that depleting 4E-T from HeLa cells significantly increased steady-state translation, in part at least due to partial relief of microRNA-mediated silencing.

## METHODS AND MATERIALS

### Plasmids

Plasmids of *Drosophila* Cup (8), CG32016 and human 4E-T (20) were gifts from Craig Smibert, Daniel St Johnston and Reinhard Luhrmann, respectively. Primers specified in Supplementary Table S1 were used to amplify their ORFs into appropriate mammalian vectors, typically in frame with either GFP or HA/NHA. In the former case, the ORFs were subcloned into EGFP-C1 and EGFP-N1 vectors (Clontech). While in general, the pattern of localization was similar for the constructs in both vectors, EGFP-C1 vectors showed a higher level of transfection and hence were used in all reported experiments. For the tethering constructs, the ORFs were first subcloned into pCI-HA or pCI-λNHA (36), using XbaI and NotI sites and subsequently subcloned as a polymerase chain reaction (PCR) product with upstream HA-tag or λNHA-tag into EGFP-C1 vector, from which GFP ORF was cut out using NheI and XmaI. Control firefly luciferase (*Photinus pyralis* in pGL3) and *Renilla* luciferase (*Rotylenchulus reniformis*; phRL-TK) with and without BoxB hairpins were described previously (36). The let-7 sites in the HCV IRES *Renilla* luciferase let-7 reporter plasmid in pRL-SV40 (37) were replaced with five BoxB elements by PCR using the *Renilla* BoxB plasmid as template. FLAG-CNOT1 and FLAG-CNOT7 plasmids were reported previously (38,39). Protein coding regions were amplified by PCR and cloned into either yeast pGADT7 AD or pGBKT7 vectors (BD Biosciences-Clontech) using the NdeI and XmaI sites. All PCR was performed with Pfu polymerase using standard conditions. Mutagenesis was carried out using the QuickChange system (Stratagene), with primers specified in Supplementary Table S1. All cloning and mutagenesis was verified by sequencing.

### Cell culture and immunofluorescence

HeLa cells were grown in DMEM+GlutaMAX (Gibco) with 10% fetal bovine serum. Cells were plated on a 20-mm coverslip in 35-mm plates (Thermo Scientific). Transfection was performed 24 h after the cells were seeded, using 4 µl of Lipofectamine 2000 (Invitrogen) and 2 µg of DNA constructs. Twenty-four hours after transfection, cells were fixed with 4% paraformaldehyde for 20 min at room temperature, permeabilized using 0.5% Triton X-100 (Sigma) and then incubated with primary antibodies followed by staining with the appropriate Rhodamine-Red-X-conjugated (1:1000) (Jackson ImmunoResearch) secondary antibodies. Primary antibodies and dilutions were used as follows: 4E-T (1:200) (Abcam), eIF4E (1:1000) (Santa Cruz), rck/p54 (1:1000) (Bethyl Laboratories). To visualize the nucleus, cells were also stained with DAPI (1.25 µg/ml) for 10 s. Leptomycin B (in methanol) (Sigma) was used at a final concentration of 5 ng/ml for 5 h. Simultaneously, the same volume of methanol was added to the control cells. Coverslips were mounted with CitiFluor for visualization under the fluorescent Zeiss AxioImager M1 microscope or Leica SP5 confocal microscope. Each sample was analysed in triplicate. At least 100 transfected cells were analysed per sample.

### Yeast two hybrid assay

pGBK-protein constructs were transformed into *Saccharomyces cerevisiae* AH109 cells as follows: 5 µl of salmon testes ssDNA (10 mg/ml), previously heated to 95°C for 10–20 min and cooled on ice, 100 µl of One-step-buffer (200 mM LiAc, 40% w/v PEG, 100 mM DTT), one yeast colony of ∼2 mm diameter and 0.5 µl of plasmid DNA (50–250 ng) were mixed by vortexing and incubated at 45°C, 30 min before plating on selective medium (minimal SD base supplemented with 2% glucose and the required dropout solution, -Trp in this case). Plates were incubated at 30°C for 3–5 days until colonies were 2–3 mm in diameter. A single colony was selected and re-streaked onto a fresh plate. These singly transformed strains were used as a yeast stock in which to transform the pGAD-protein constructs. The second round of transformation was performed in exactly the same way as described above with the exception that selection required –Trp-Leu dropout medium to select for both plasmids. Stocks were made from single colonies of double transformants and re-streaked on both –Trp-Leu and –Trp-Leu-Ade-His dropout plates to screen for plasmid retention and high-stringency protein interactions, respectively.

### GFP-Trap immunoprecipitation

GFP-Trap® Magnetic beads (Chromotek) (25 µl) were resuspended in 250 µl of ice-cold NET buffer (50 mM Tris–HCl, pH 7.5, 150 mM NaCl, 0.5% v/v NP-40, 1 mM EDTA, 0.25% w/v gelatine, 0.02% v/v sodium azide) supplemented with complete proteinase inhibitor cocktail (Roche) in hydrophobic tubes. Beads were washed by magnetic separation and lysates added (1000 µg in 1 ml NET buffer). Mixtures were incubated for 1.25 h at 4°C with constant rotation. Supernatants were removed after magnetic separation of samples, beads washed and protein eluted in 30 µl 1× SB. Fifteen microliters of input (40 µg) and output samples were loaded onto 15% SDS-PAGE or gradient gels and assessed by western blotting.

### SDS-PAGE and western blotting

Our standard 15% SDS-PAGE and western blotting procedures have been described previously (40). On occasion, as stated, gradient gels (NuPAGE® Novex® 4–12% Bis-Tris Gel, Life Technologies™) were used. Blots were developed using ECL and the primary antibodies and dilutions were used as follows: 4E-T (1:200) (Abcam), eIF4E (1:1000) (Santa Cruz), Flag (1:1000) (Sigma), GFP (1:1000) (Santa Cruz), HA (1:2000) (Roche), rck/p54 (1:1000) (Bethyl Laboratories) and tubulin (1:2000) (Abcam).

### Dual-luciferase tether function assay

HEK293 cells were grown in DMEM+GlutaMAX (Gibco) with 10% fetal bovine serum. Cells were plated on 35-mm plates (Thermo Scientific). Transfection was performed 24 h after the cells were seeded, using 3 µl of Lipofectamine 2000 (Invitrogen) and 1 µg of DNA constructs encoding GFP, Pat1b, 4E-T WT and mutants fused to either HA-tag (control) or λNHA, 5 ng of *Renilla* luciferase reporter mRNA with 5 boxB sites and 100 ng of control firefly luciferase reporter mRNA. When DNA amounts between constructs were varied (to achieve equivalent expression levels), they were substituted by respective amounts of pcDNA3 plasmid to make up 1 µg of DNA. After 48 h, cells were harvested and split between two tubes and used for either protein or RNA extraction. Protein extraction was performed using 1× Passive Lysis Buffer (Promega) on a rocker for 15 min at room temperature. Each sample was analysed in triplicate. The luciferase assay was performed as described (41), using the Dual-luciferase Assay System (Promega) and the Glomax 96 microplate luminometer. Levels of tethered proteins were optimized by varying transfected DNA concentrations, to give approx equal protein concentrations between HA/NHA pairs, and between different mutants/truncated versions, as verified with western blotting using HA antibodies.

### RNA extraction and quantitative PCR

RNA was extracted with Trizol (Invitrogen) following the manufacturer’s instructions and treated two times with RQ1 DNase I (Promega). RNA concentration was quantified using Nanodrop (Nanodrop Technologies). Two micrograms of RNA were converted to cDNA using primers specified in Supplementary Table S1 and AMV reverse transcriptase (Promega). Quantitative RT-PCR reactions were performed using SYBR Green I (Sigma) mix in a Rotor-Gene 6000 (Corbett research) with the following cycling conditions: 95°C, 10 min; 40 × (95°C, 10 s; 55°C, 15 s; 72°C, 20 s). To confirm specificity, amplification was followed by melting temperature analysis and agarose gel electrophoresis. In addition, minus reverse transcriptase controls were included to rule out contamination by genomic DNA. The relative change in mRNA levels was analysed using Rotor-Gene 6000 software package (Corbett Research/Qiagen). The fold change in mRNA levels was calculated relative to the control and normalized to GAPDH mRNA in triplicates in three independent experiments.

### Northern blotting

RNA was extracted using the RNAeasy kit (Qiagen) according to the manufacturer’s instructions. RNA was denatured with glyoxal before electrophoresis on a 1.1% agarose gel and blotted overnight on to Hybond-N (Amersham) according to manufacturer’s instructions. The labelling probe was prepared using Amersham megaprime DNA Labelling System (GE Healthcare) and deoxycytidine 5-triphosphate, [α-^32^P] (Perkin Elmer). Probes corresponded to the full-length open reading frames, obtained by restriction digests and gel extraction (Qiagen kit). Hybridization of the probe was performed overnight in 5× SSC, 5× Dedhardt’s, 0.5% SDS, 0.1% sodium pyrophosphate, 100 µg/ml heparin solution at 65°C. The blot was washed 2 × 20 min at 60°C. Quantification was performed using Molecular Dynamics Storm 840 and ImageQuant software. Individual bands were measured and the background (an equal area either above or below each band) was substracted. GAPDH RNA was used as a loading control.

### RNAi interference and let-7 luciferase assay

For siRNA knock-down experiments, Dharmacon control siRNA 3 was obtained from Thermo Fisher. Ambion silencer select siRNAs against 4E-T [s32162 (no.1) and s32161 (no. 2)] and LSm1 were obtained from Life Technologies, and TNRC6A/B siRNAs were obtained from Life Technologies (TNRC6A-s26154; TNRC6B- s23060). HeLa cells were grown in DMEM with 10% FBS, with 30 000 cells seeded per well in 24-well plate format. siRNA (30 nM) was transfected with Dharmafect 1 transfection reagent (Thermo Fisher) as per manufacturer’s instruction. Subsequent transfection of luciferase reporter plasmids were carried out using Lipofectamine 2000 (Life Technologies) 48 h after siRNA transfections were performed as described (42). After a further 24 h, cells were lysed with passive lysis buffer (Promega) and luciferase activity was measured with Promega dual luciferase reporter assay system. The relative luciferase activity of each well was obtained by dividing the activity of *Renilla* luciferase, with or without let-7 miRNA target sites, against the activity of firefly luciferase, which served as transfection control for each well. Data were collected from three independent experiments; error bars represent pooled standard deviation of each biological repeat. siRNA knock-down was assessed by western blotting using 4E-T (Abcam), Lsm1 (BenewayBio), TNRC6A (Novus Biologicals), HMGA2 (Abcam), GAPDH (Santa Cruz) and vinculin (Abcam) antibodies.

### ^35^S methionine incorporation

To determine the rate of new protein synthesis in control and 4E-T knock-down cells, HeLa cells were transfected with siRNA as described previously for 72 h. The final cell confluency did not exceed 80% in all cases. Medium was washed twice with phosphate buffered saline and incubated with methionine-free DMEM (Sigma-Aldrich) supplemented with 30 μCi/ml ^35^S methionine/cysteine (Hartmann Analytic) for 45 min. Medium was removed after incubation and cells were washed twice with ice cold phosphate buffered saline before lysed with Passive Lysis Buffer (Promega). To determine the incorporation of ^35^S methionine isotopes, cell lysates were then precipitated with trichloroacetic acid precipitation on Whatman filter paper, while the protein content of each sample was determined with the Bradford protein assay (Bio-Rad). Data were collected from three independent experiments; error bars represent pooled standard deviation of each biological repeat.

## RESULTS

### Bioinformatic and localization analyses identify the *Drosophila* homologue of h4E-T

In human tissue culture cells, 4E-T localizes to P-bodies and is required for P-body integrity (20,21,43). To obtain initial hints regarding the region that promotes 4E-T accumulation in P-bodies, we performed multiple protein sequence alignments of 9 vertebrate 4E-T proteins (*Danio rerio*, *Oryzias latipes*, *Xenopus laevis*, *Xenopus tropicalis*, *Bos taurus*, *Rattus rattus*, *Mus musculus*, *Homo sapiens* and *Gallus gallus*) using ClustalW and Emboss Needle programs. Alignments between the vertebrate 4E-T proteins revealed a high degree of conservation throughout their sequences (for example, human and mouse proteins share 90% identical residues and 95% similar ones), which did not allow us to focus on any particularly conserved regions.

**Figure 1.**
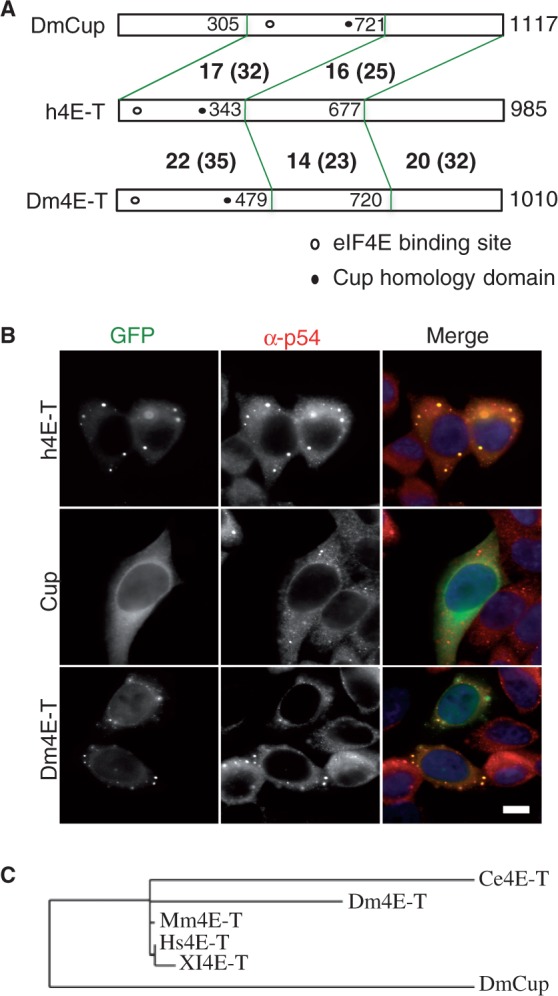
*Drosophila* CG32016 is closer in sequence and cellular localization to human 4E-T than to Cup. (**A**) Cartoon of human 4E-T, *Drosophila* CG32016 (Dm4E-T) and Cup proteins, indicating degree of conservation between N-terminal third, middle third and C-terminal third regions (% identical, and similar residues in brackets according to EMBOSS needle program). Unfilled circle indicates the approximate location of the canonical eIF4E-binding site, and the filled circle that of the Cup homology domain. (**B**) Fluorescence imaging of HeLa cells transfected with GFP-4E-T, GFP-Cup and GFP-Dm4E-T/CG32016, also stained with antibodies against the P-body marker protein rck/p54, and with DAPI. Scale 10 µm. (**C**) Phylogeny tree of human (NM_001164501), mouse (BC033410), *X. laevis* (NM_001093241, *C. elegans* 4E-T (Spn-2, NM_065508), *Drosophila* Cup (CG11181, NM_078769) and 4E-T (CG32016; NM_166798) proteins according to phylogeny.fr (44). Branch length is proportional to the number of substitutions per site.

Therefore, we expanded the analysis by including *Drosophila melanogaster* Cup, which is related to h4E-T in sequence, that regulates translation by interacting with 3′ UTR-binding proteins and eIF4E, and co-localizes with Me31B (rck/p54) in cytoplasmic particles in nurse cells (see ‘Introduction’ section). However, interestingly, despite their apparent common role in translational control, they do not share high sequence homology (13% identical, 22% similar residues overall); except for the canonical eIF4E-binding site and a region located towards the N-terminus of h4E-T and more centrally in Cup, which we named the Cup Homology Domain (CHD) (Figure 1A, Supplementary Figure S1B). In addition, both proteins have C-terminal stretches of Q residues, particularly notable in Cup.

To further investigate the degree of functional conservation between human 4E-T and Cup, we compared their subcellular localization when tagged with GFP in human tissue culture cells. To this end, the full length Cup cDNA sequence was cloned into the EGFP-C1 vector and protein localization was examined using immunofluorescence microscopy. Cells were immunostained with rck/p54 antibodies as a marker for endogenous P-bodies. In contrast to h4E-T-GFP, which localized to P-bodies in >90% of cells, as well as being free in the cytoplasm mirroring the endogenous protein distribution, Cup-GFP was found diffused in the cytoplasm with weak nuclear localization, but showing no enrichment in P-bodies (Figure 1B).

That *Drosophila* Cup did not behave like 4E-T in HeLa cells was to an extent surprising, since other related proteins such as Pat1b and HPat localize to P-bodies in HeLa and S2 cells, respectively (40,45), but could simply reflect the lack of appropriate fly cofactors in mammalian cells. Nevertheless, these observations prompted us to examine a Cup-related fly protein, the product of the poorly characterized CG32016 gene (hereafter named Dm4E-T), which is expressed in all larval and adult organs and tissues, with highest levels in the ovary (FlyBase Encode). Human 4E-T and Dm4E-T share 20% identical and 32% similar residues, with the most conserved regions in the N-terminal third of the proteins, and the C-terminus. In contrast, the most conserved region between h4E-T and Cup lies in the central third of Cup, which contains the eIF4E-binding site and the CHD (Figure 1A; Supplementary Figure S1). Strikingly, unlike Cup, Dm4E-T-GFP showed P-body localization in the majority of transfected HeLa cells (Figure 1B), as well as diffuse cytoplasmic and weak nuclear localization. Moreover, Leptomycin B treatment of the transfected cells, to prevent Crm1-dependent nuclear export (46), showed that Dm4E-T, as well as human 4E-T, are nucleocytoplasmic shuttling proteins, with full nuclear retention observed in >80% of transfected cells, whereas DmCup remained partitioned between cytoplasm and nuclei in treated cells (Supplementary Figure S2). Phylogenetic analysis confirmed that Dm4E-T (CG32016) and *C. elegans* Spn-2 (10) are more closely related to human 4E-T than to *Drosophila* Cup (Figure 1C). Altogether we conclude that DmCG32016 (Dm4E-T) is the homologue of mammalian 4E-T, based on its sequence and cellular distribution.

### The 4E-T C-terminus, downstream of a.a. 694, is required for its localization to P-bodies

To identify the regions of human 4E-T that mediate its P-body localization, we examined the cellular localization of 4E-T-GFP protein variants in HeLa cells, truncated from either the N- or the C-terminus. To define where to truncate 4E-T, we used the multiple sequence alignment of human, *X. laevis* and *Drosophila* 4E-T and *C. elegans* Spn-2 proteins generated by the T-COFFEEE advanced programme and ClustalW, secondary structure prediction (using Jpred 3 and PSIPRED), domain predictions (using DOMAC) and prediction of order and disorder regions (using POODLE). The protein sequence was truncated before or after the conserved regions identified in this search (Supplementary Figure S1C).

**Figure 2.**
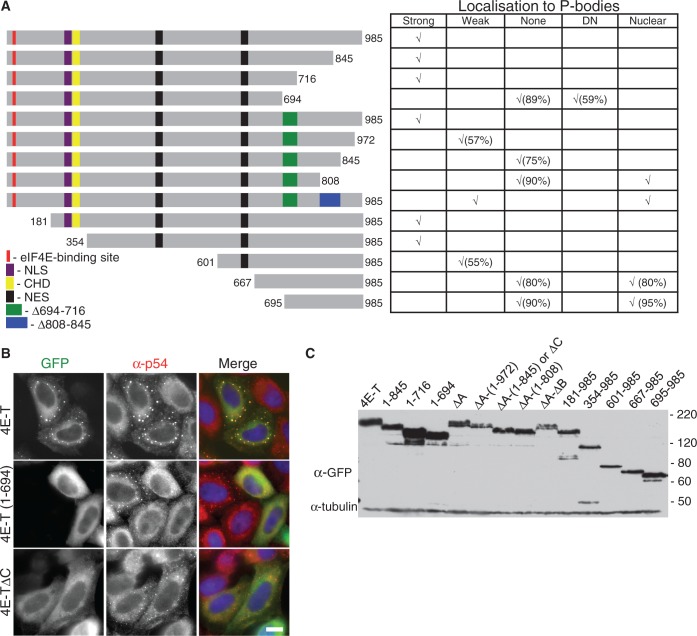
The C-terminus of 4E-T promotes its localization in P-bodies. (**A**) Summary scheme of N- and C-terminal truncated constructs of GFP-4E-T, with table indicating localization of truncated proteins to P-bodies, or to nuclei. Strong localization indicates localization in a large number of foci in 80% of cells or more, weak localization indicates localization in fewer, smaller foci. DN is dominant negative for endogenous P-bodies. (**B**) Fluorescence imaging of HeLa cells transfected with full-length and indicated truncated versions of GFP-4E-T, also stained with antibodies against the P-body marker protein rck/p54, and with DAPI. Scale, 10 µm. (**C**) Western blotting analysis of HeLa cells transfected with indicated 4E-T proteins developed with GFP and tubulin antibodies, as loading control.

We initially tested C-terminally truncated proteins, with an intact eIF4E-binding site. The mutants truncated downstream of amino acids 845 and 716 localized to P-bodies as well as the full-length protein (Figure 2A). In contrast, 4E-T truncated at 694 a.a. was dispersed in the cytoplasm in >89% of transfected cells, and in the remaining 11% of the cells, it localized weakly to P-bodies in the sense that they were smaller and fewer compared with full-length 4E-T (Figure 2A). Moreover, this truncation affected P-body integrity in a dominant-negative manner, as 59% of transfected cells did not have detectable endogenous P-bodies (Figure 2B).

These results suggested that the region spanning 694–716 a.a. in 4E-T, conserved in vertebrate homologues, as well as in *Dm*4E-T and *Ce*Spn2, but absent from Cup, is important for P-body localization. We thus tested the effect of deleting it from the full-length sequence, but surprisingly, 4E-T-Δ694-716 (ΔA segment; green in Figure 2A) still strongly localized to P-bodies, similarly to wild-type 4E-T, demonstrating that the deleted region contributes to but is not absolutely required for localization (Figure 2A). Thus, we speculated that in the absence of the A sequence, a domain downstream of 716 a.a. may play a redundant role in P-body localization.

To test this, we examined the GFP-4E-T-ΔA protein truncated at a.a. 972 and 845. Both these mutants localized less efficiently to P-bodies than the parent GFP-4E-T-ΔA protein (Figure 2A). GFP-4E-T-ΔA-(1-972) was detected in P-bodies in ∼57% of cells, though P-bodies were smaller and usually fewer compared with those detected with GFP-4E-T WT and -4E-T-ΔA. At least 75% of cells transfected with 4E-T-ΔA-(1-845) did not contain any GFP P-bodies, with the remaining cells having significantly smaller and fewer foci compared with the full-length protein. However, 75% of the cells contained endogenous P-bodies, showing that 4E-T-ΔA-(1-845) (renamed 4E-T-ΔC) does not strongly affect P-body integrity (Figure 2B, Supplementary Figure S3). Further C-terminal truncations [4E-T-ΔA-(1-808) and 4E-T-ΔA-Δ(808-845)] did not localize to P-bodies in >80% of transfected cells, but also showed partial nuclear localization and were not used further.

To validate these results, we investigated 4E-T-GFP proteins lacking N-terminal regions. Truncated mutants whose ORFs started at a.a. 181 and 354 retained their ability to localize to P-bodies, similarly to full-length 4E-T, confirming that C-terminal sequences were required for localization while eIF4E-binding was not (Figure 2A). 4E-T-(601-985) showed weaker P-body localization, indicating the contribution of additional residues upstream of residue 601. However, as 4E-T-(667-985) and 4E-T-(695-985) proteins localized to nuclei in ∼ 80 and 95% of cells, respectively (Figure 2A), it was not possible to determine the minimal sufficient sequence for P-body localization. All mutant proteins were expressed to similar extents, as shown by western blotting with anti-GFP antibodies (Figure 2C).

These results show that the sequences necessary for 4E-T residency in P-bodies are located in its C-terminus, downstream of a.a. 694. Moreover, two types of localization defective mutants were obtained—permissive [4E-T-ΔC; in other words 4E-T-Δ694-716-(1-845)] and nonpermissive (4E-T 1-694) for endogenous P-bodies.

### 4E-T recruits endogenous eIF4E to P-bodies via C-terminal sequences and YXXXXLL

**Figure 3.**
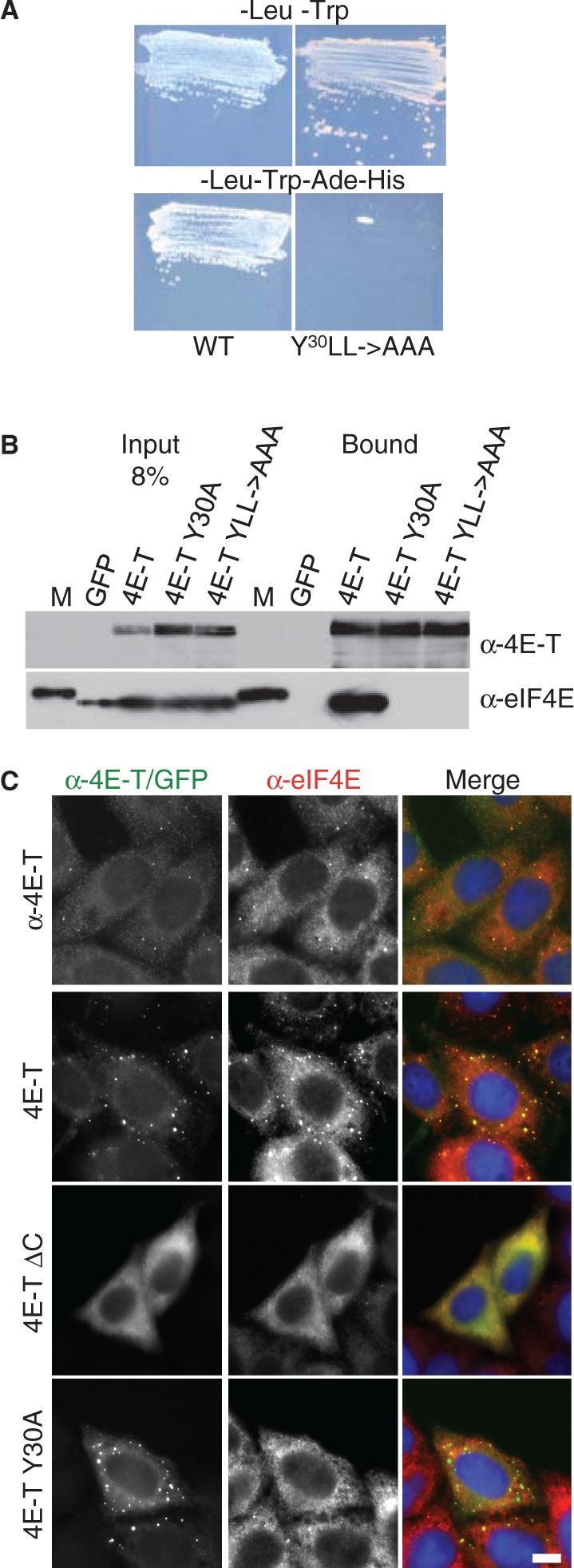
4E-T recruits eIF4E to P-bodies via Y30XXXXLL and its C-terminal localization sequence. (**A**) 4E-T binds eIF4E in the yeast two hybrid system via YXXXXLL. Growth in –Leu-Trp drop-out media to select for plasmids, and in –Leu-Trp-Ade-His to select for interactions. (**B**) GFP-Trap co-immunoprecipitation of eIF4E in HEK293 cells with indicated GFP-4E-T proteins analysed by western blotting with 4E-T and eIF4E antibodies. Input and bound proteins were compared. M is a molecular weight protein standard. (**C**) Fluorescence imaging of untransfected HeLa cells stained with 4E-T and eIF4E antibodies (top row), and of cells transfected with wild-type and mutant GFP-4E-T proteins (2nd to 4th row), also stained with eIF4E antibodies, and with DAPI. Scale bar, 10 µm.

We then wished to test the effect of wild-type and mutant 4E-T proteins on endogenous eIF4E distribution. First, using the yeast two hybrid assay, we verified the interaction between human 4E-T and eIF4E proteins. Mutagenesis of the three key residues of the eIF4E-binding site YXXXXLL led to complete loss of binding in yeast (Figure 3A). Similarly, using the GFP-Trap pull-down assay (see ‘Materials and Methods’ section) with lysates prepared from transfected HEK293 cells, mutation of the critical tyrosine alone (Y30A) or in combination with the two leucine residues (YLL → AAA), referred to as 4Emut, prevented the robust binding of eIF4E to 4E-T (Figure 3B). Next, we showed that recruitment of eIF4E to P-bodies by 4E-T requires its P-body localization domain, arguing that this recruitment is a direct effect of ectopic 4E-T. In untrasfected cells, eIF4E detected with a mouse monoclonal antibody was found largely diffuse in the cytoplasm, with enrichment in a few foci (Figure 3C, top panels). 4E-T-GFP redistributed a significant fraction of cytosolic eIF4E to P-bodies, but the mutant version ΔC, which does not localize to P-bodies (Figure 2B), had no significant effect on eIF4E distribution (Figure 3C, middle panels). As predicted, and in line with previous studies (21,22), eIF4E distribution observed by both fluorescent and confocal microscopy was unaltered by GFP-4E-T-Y30A, though this localized to P-bodies as efficiently as the wild-type protein (Figure 3C, Supplementary Figure S3).

### Tethered 4E-T represses translation, in a P-body-independent manner

**Figure 4.**
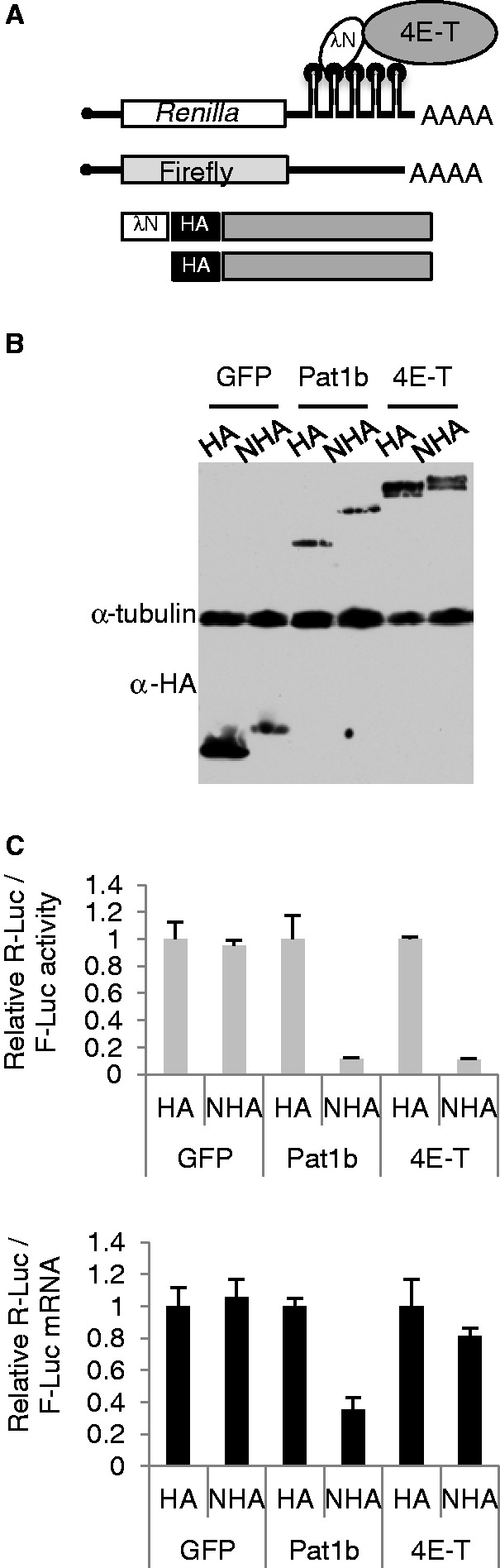
4E-T represses translation of tethered mRNA. (**A**) Schematic cartoon of the tether function assay. (**B**) Western blot analysis of HEK293 cells transfected with HA/NHA GFP, Pat1b and 4E-T plasmids, developed with HA and tubulin antibodies. (**C**) Ratios of Renilla and firefly luciferase activities (top) and mRNAs (relative to GAPDH) (bottom) for HA/NHA-GFP, -Pat1b and -4E-T, with the HA results set to 1.

Next, we used the tethered function assay to ask what effect recruiting wild-type and mutant 4E-T to the 3′ UTR of a reporter mRNA had on its expression and stability. We used the λN-BoxB system, with *Renilla* luciferase as the test mRNA with five 3′ UTR BoxB hairpins and firefly luciferase as the co-expressed control mRNA, lacking 3′ UTR elements. 4E-T expression constructs, and control constructs, contained a combined λN-HA (NHA) element or an HA tag alone upstream and in frame with the main ORF (Figure 4A). Typically, the two luciferase plasmids and one of the 4E-T proteins or control protein plasmids were co-transfected into HEK293 cells for these experiments, to benefit from their higher transfection efficiency. The levels of expression of these HA/NHA proteins were adjusted to be approximately constant, as monitored by western blotting with HA antibodies (based on data in Supplementary Figure S6, discussed in further detail below). After 48 h, lysates were prepared and assayed in the Dual Luciferase assay system, and RNA was extracted for qPCR of reporter mRNA levels, relative to GAPDH. Results are usually reported as ratios of RLuc/FLuc activities and mRNA levels in the presence of HA-tagged or NHA-tagged proteins.

First, we compared the effects of tethering 4E-T to that of two control proteins, the P-body protein Pat1b and GFP (Figure 4B). Previously, tethered Pat1b was shown to result largely in the decay of bound mRNA in mammalian cells (47,48). Both NHA-Pat1b and NHA-4E-T reduce normalized luciferase activity, relative to their HA versions, and to the two GFP proteins (Figure 4C). In an additional specificity test, we demonstrated that the effect of NHA-4E-T on tethered mRNA required the BoxB hairpins in the 3′ UTR of Rluc mRNA (Supplementary Figure S4). qPCR of the reporter mRNA levels showed that NHA-4E-T largely reduces expression of tethered mRNA at the level of translation (Figure 4D), in contrast to Pat1b, which reduced both translation and mRNA levels, in line with previous studies using MS2 and PP7 tethering systems rather than λN (47,48).

**Figure 5.**
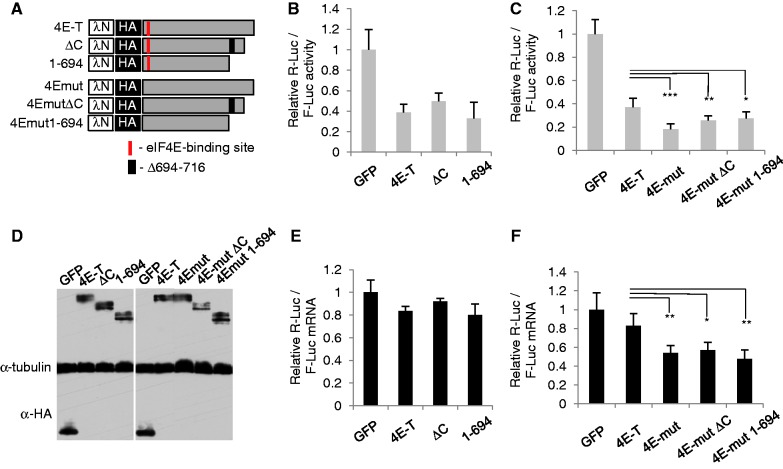
4E-T represses translation of tethered mRNA in a P-body and eIF4E-independent manner. (**A**) NHA-GFP, and NHA-4E-T wild-type and mutant proteins as indicated were transfected into HEK293 cells alongside Renilla-BoxB and control firefly luciferase plasmids. Mutants in P-body localization (**B** and **E**), and eIF4E binding (**C** and **F**) were assessed. (B and C) The relative levels of luciferase activities. (E and F) The relative levels of luciferase mRNAs, normalized to GAPDH. *P < 0.05; **P < 0.01, ***P < 0.001, t-test. (**D**) Western blot analysis of transfected cell lysates developed with HA and tubulin antibodies.

Next we asked whether P-body localization was involved in the translational repression by 4E-T, by contrasting the wild-type protein with the localization impaired mutants (Figure 5). Both NHA-4E-T ΔC and NHA-4E-T(1-694) repressed translation (rather than enhancing decay) of bound mRNAs as effectively as the full-length protein relative to NHA-GFP (Figure 5B and E), suggesting that P-body localization of the tethering protein was not involved in repression, and also that the integrity of endogenous P-bodies was not important for this silencing mechanism.

### 4E-T represses bound mRNA in an eIF4E-independent manner

Next, we wished to investigate the effect of preventing eIF4E binding to tethered 4E-T on its ability to repress translation. The previous experiments indicated that control mRNA (FLuc) activities were reduced in cells transfected with HA/NHA-4E-T, though not by HA/NHA-GFP or -Pat1b proteins, while bound mRNA (RLuc) was significantly more inhibited only by NHA-4E-T and NHA-Pat1b (data not shown). Moreover, in ^35^SMet incorporation assays, we saw a reduction (∼50%) in global protein synthesis in the presence of GFP-4E-T but neither by GFP alone nor the Y30A 4E-T mutant (Supplementary Figure S5). We provisionally concluded that ectopic 4E-T has two effects on protein synthesis, a general one affecting all translation, presumably by sequestering eIF4E, and an additional one on bound mRNAs. To assess this more thoroughly, we transfected three concentrations of NHA-tagged protein plasmids, which resulted in a wide range of expression as shown by western blotting (Supplementary Figure S6). As expected, NHA-GFP and NHA-Pat1b, irrespective of levels, have little effect on the control firefly luciferase activity, while NHA-4E-T reduces its expression in a dose-dependent manner, as does NHA-4E-T ΔC, which contains the eIF4E-binding site but lacks the P-body localization sequence (Supplementary Figure S6). Moreover, both NHA-4E-T and NHA-Pat1b significantly reduce the expression of *Renilla*-BoxB mRNA, in contrast to NHA-GFP. The panel showing the luciferase activity ratios confirms the previously reached conclusion that 4E-T and Pat1b both reduce expression of bound mRNA (Supplementary Figure S6). By reporting the separate luciferase data, we further verify that 4E-T, unlike Pat1b, has both a general effect on protein synthesis translation, as shown by others (21,22), and an additional repressive effect when tethered.

We then tested the effect of mutating the 4E-T eIF4E-binding site YX_4_Lϕ on translation, having previously verified loss of eIF4E binding using GFP-Trap immunoprecipitation (Figure 3). As expected, preventing eIF4E binding in 4E-T abolished its inhibition of control FLuc activity, but, surprisingly, the mutant was able to reduce Rluc activity (Supplementary Figure S6). In a subsequent experiment in which comparable levels of 4E-T proteins were expressed, the ratio of Rluc/Fluc activities (which in effect cancels out the general inhibitory effect of unbound 4E-T on both luciferase mRNAs) indicates that the eIF4E-binding mutant of 4E-T represses bound mRNA at least as well as the wild-type protein, if not significantly more (Figure 5C). We also noted that NHA-4EmutΔC and -1-694, which both neither bind eIF4E nor localize to P-bodies efficiently, repress translation of bound mRNA similarly to the NHA-4Emut protein. Moreover, all three 4Emut proteins resulted in modest reduction in bound mRNA levels relative to the wild-type protein, presumably responsible for the decrease in tethered mRNA activity (Figure 5F). We conclude that 4E-T represses bound mRNA translation independently of interacting with eIF4E, while general protein synthesis is only affected by 4E-T that sequesters eIF4E.

### The N-terminal 1-180 4E-T fragment does not repress bound mRNA

**Figure 6.**
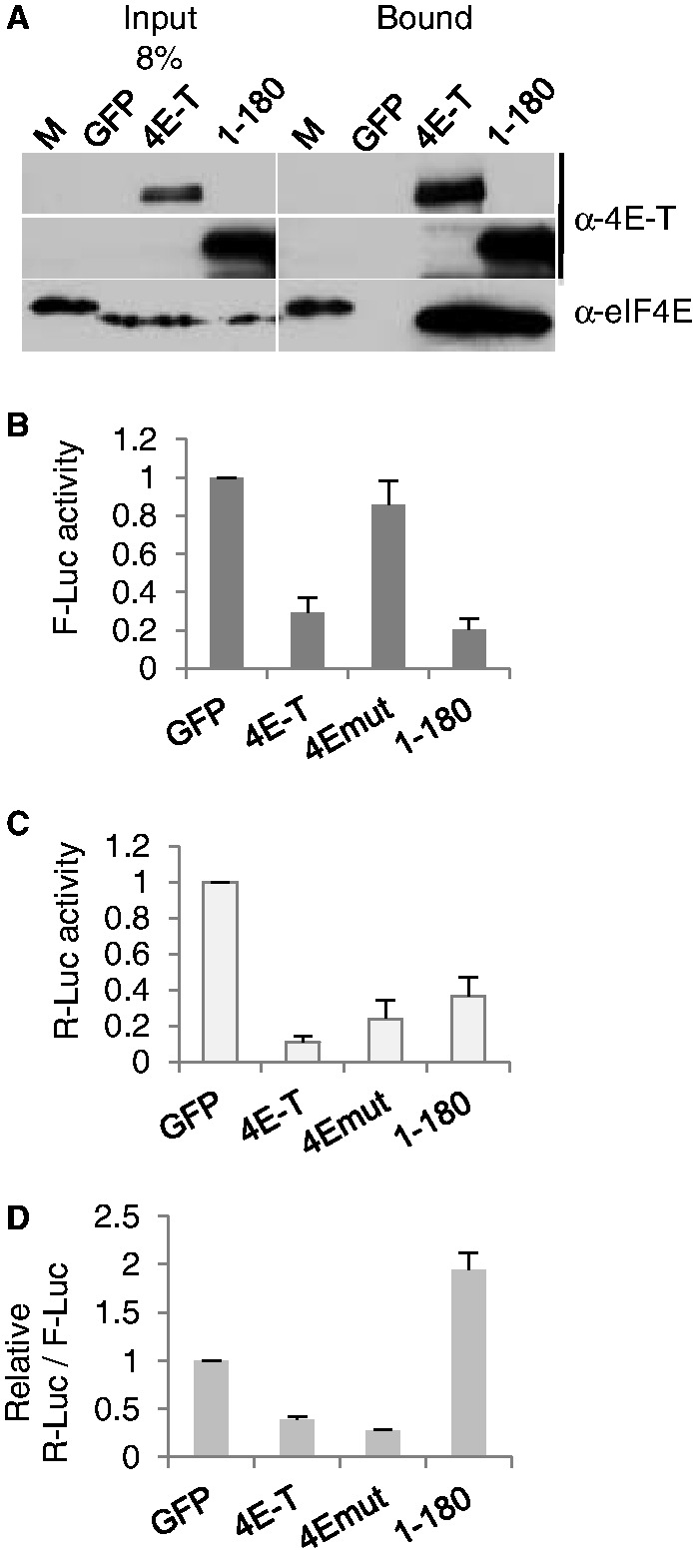
Tethering the N terminal 1–180 fragment, which binds eIF4E does not repress translation of bound mRNA. (**A**) GFP-Trap co-immunoprecipitation of eIF4E in HEK293 cells with GFP alone, GFP-4E-T and GFP-4E-T1-180 proteins analysed by western blotting with 4E-T and eIF4E antibodies. Input and bound proteins were compared. M is a molecular weight protein standard. (**B–D**) NHA-GFP, and -4E-T wild-type, eIF4E-binding mutant (Y30A) and 1–180 proteins as indicated were transfected into HEK293 cells alongside luciferase plasmids, and the level of firefly (B) and Renilla luciferase (C) activities was determined; with panel D showing their relative activities.

To independently assess the role of eIF4E binding in 4E-T translational repression, we cloned the N-terminal 1–180 fragment of 4E-T downstream of NHA. This fragment contains the eIF4E-binding site and binds eIF4E in a pull-down assay (Figure 6A) but lacks both NLS and NES sequences (Figure 2A). In the tether function assay, NHA-4E-T1-180 behaves as the full-length 4E-T protein in terms of its effects on general protein synthesis, as expected, but does not additionally repress bound Rluc mRNA (Figure 6 and Supplementary Figure S6). NHA-4E-T-Y30A served as an eIF4E-binding mutant control in this experiment, which did not affect Fluc mRNA activity but repressed tethered mRNA (Figure 6B and C resp.). Thus tethering a protein fragment capable of binding eIF4E does not promote repression, further confirming the previous findings that 4E-T does not need to interact with eIF4E to repress bound mRNA.

To further delineate the regions required for repression, we also subcloned a Mid-domain (180–601) containing the conserved CHD, and the C-terminal (602–985) domain encompassing the P-body localization signals. Neither of these domains affected bound mRNA translation significantly, though unlike the N-terminal fragment, the Mid- and C-ter domains were poorly expressed (data not shown). We note, however, that since tethered 4E-T repression is relatively protein concentration independent (Supplementary Figure S6), any possible independent effects of the subdomains should have been detected, despite their low levels. To explore the possible role of the CHD, we removed this ∼ 25-amino acid-long highly conserved region from 4E-T (Supplementary Figure S1B), and found that it repressed tethered mRNA as well as the full-length protein (Supplementary Figure S7). As 4E-T 1-694 repressed bound mRNA equally efficiently (Figure 5C), altogether we conclude that N- and Mid-domains need to be contigous to exert repression. Moreover, the CHD does not appear to play a significant role in the silencing mechanism, with the caveat that it may provide a binding site for a 3′ UTR-binding protein, functionally replaced in the tethering assay by the lambda N peptide.

### eIF4E binding stabilizes bound mRNA

**Figure 7.**
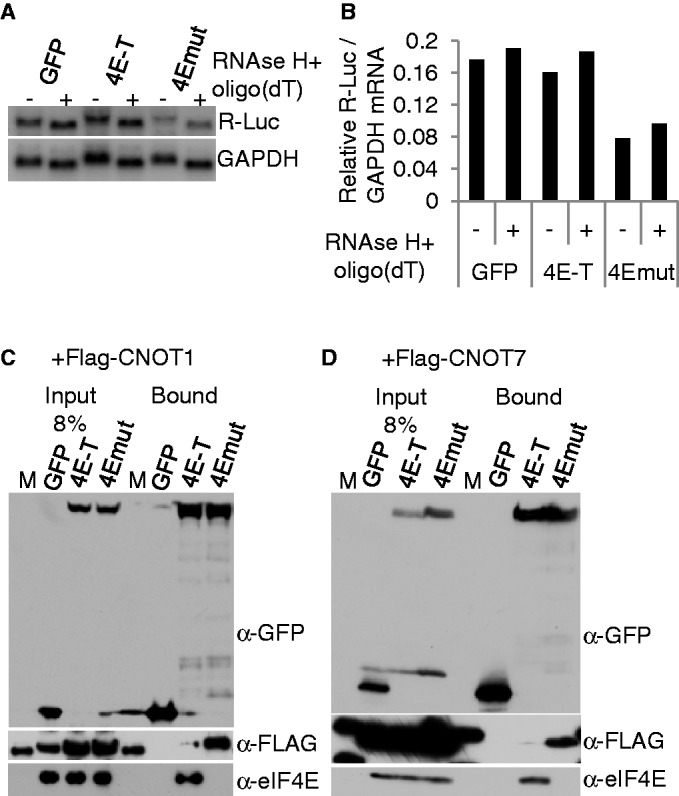
Tethered 4E-T does not promote deadenylation of bound mRNA, while loss of eIF4E binding results in modest mRNA decay, involving enhanced binding of CNOT1/7 deadenylase subunits. (**A**) Northern blot analysis of RNA samples from HEK293 cells transfected with tethering plasmids including NHA-GFP, -4E-T and -4E-T proteins mutant in eIF4E-binding (4Emut, YLL → AAA). RNAs were treated (+) or not (−) with oligo(dT) and RNAse H, and the blot was hybridized with a Renilla luciferase probe, and a GAPDH probe as loading control. (**B**) Phosphoimager quantitation of northern blot shown in A. (**C** and **D**) HEK293 cells were co-transfected with GFP, GFP-4E-T or GFP-4Emut (Y30A) and either FLAG-CNOT1 (C) or FLAG-CNOT7 (D) and lysates were immunoprecipitated with GFP-Trap and the input and bound proteins assessed by western blotting with GFP, FLAG and eIF4E antibodies. M is a molecular weight protein standard. Denaturing gradient gel used in C, standard 15% SDS-PAGE in D.

The results so far demonstrate that loss of eIF4E binding does not relieve repression of reporter mRNA by tethered 4E-T, and modestly destabilizes reporter mRNA. mRNA decay is typically initiated by deadenylation promoted by the CCR4-NOT (CNOT) complex followed by decapping and 5′ exonuclease action (49,50). To examine the polyadenylation status of reporter mRNA bound by wild-type and mutant 4E-T, we performed northern blot analysis with RNA samples extracted from cells transfected with tethering plasmids. RNA samples were left untreated, or treated with oligo(dT) and RNAse H to remove the poly(A) tail. The results show that tethered 4E-T does not significantly deadenylate reporter mRNA, compared with tethered GFP; in both cases a size shift is seen between untreated and treated samples. A similar shift is seen in the case of mutant 4E-T, which does not bind eIF4E, though we consistently noted a lower *Renilla* luciferase signal, relative to that of GAPDH, indicative of mRNA destabilization (Figure 7A and B). We thus tested the possible interaction of GFP-4E-T with the CNOT deadenylase complex, composed of ∼10 subunits including the large CNOT1 bridging subunit and two catalytic ones, CNOT6 (Ccr4) and CNOT7 (Pop2), using FLAG-tagged CNOT1 and CNOT7. In both cases, we noted a weak interaction between GFP-4E-T and the FLAG-CNOT subunit, which was considerably enhanced in the case of GFP-4Emut, which does not bind eIF4E (Figure 7C and D). Importantly, there was no major difference in expression levels of GFP-4E-T and GFP-4Emut or of their enrichment by GFP-Trap. While the underlying reason for this enhanced interaction between 4E-T and CNOT1/7 in the absence of eIF4E binding is not known, it is in line with our previously observed increase of reporter mRNA decay bound by 4Emut (Figure 5F). In summary, tethering wild-type 4E-T to reporter mRNA does not result in its deadenylation. In the case of mRNAs bound by a mutant 4E-T, which cannot bind eIF4E but shows enhanced interactions with CNOT1/7 subunits, lower steady-state levels of mRNA were observed both by qPCR and Northern blot analysis (Figures 5 and 7).

### HCV IRES overcomes translational repression imposed by bound 4E-T

**Figure 8.**
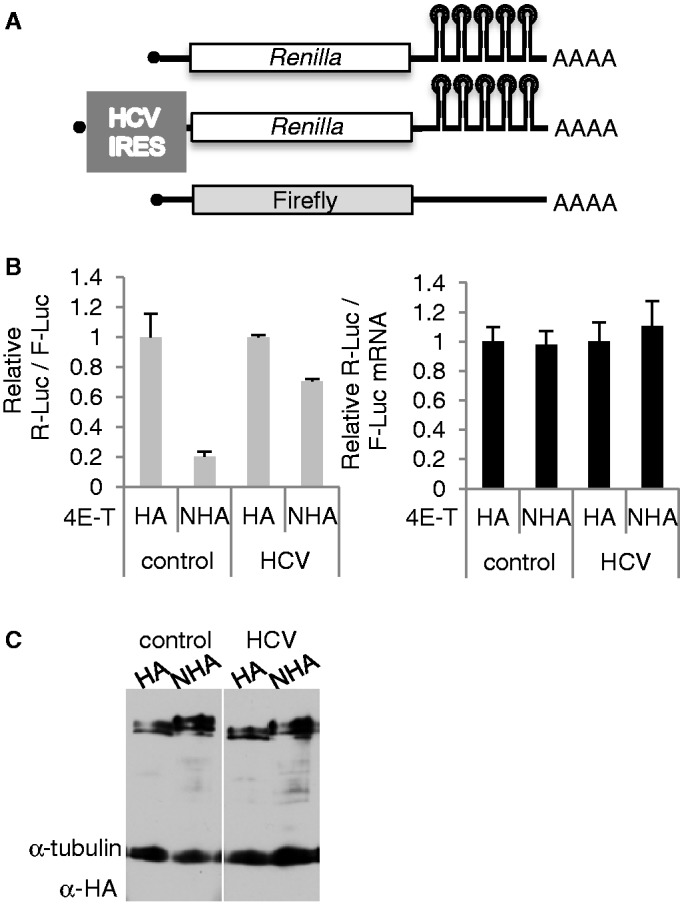
Tether function assay with IRES Renilla reporter mRNA. (**A**) HEK293 cells were transfected with plasmid DNA encoding control Rluc-BoxB mRNA or with HCV Rluc-BoxB mRNA, and firefly luciferase control plasmid alongside HA and NHA-4E-T plasmids. (**B**) Ratios of Renilla and firefly luciferase activities (left) and mRNAs (relative to GAPDH) (right) for HA/NHA-4E-T, with the HA results set to 1. (**C**). Western blot of transfected cell lysates developed with ECL and HA and tubulin antibodies.

Next we assessed whether a viral IRES would relieve the translational repression by tethered 4E-T. We used the HCV IRES, which binds eIF3 and the 40S ribosomal subunit directly to promote translation initiation, and does not require eIF4F/eIF4B or eIF1/1A (51). Plasmid Rluc DNAs with 3′ UTR BoxB motifs with and without the IRES upstream of RLuc were co-transfected with control Fluc plasmids and HA/NHA-4E-T plasmids (Figure 8A). The resulting luminometry data, expressed as a HA/NHA ratio of relative luciferase activities, showed that the HCV IRES largely overcame the repression imposed by tethered 4E-T. qPCR analysis of these samples showed that bound 4E-T does not affect the levels of either *Renilla* luciferase mRNA (Figure 8B, and Supplementary Figure S8). These results mirror those we obtained previously in *Xenopus* oocytes with microinjected *in vitro* transcribed mRNA. We found that Acapped reporter mRNAs bearing the CSFV IRES were as robustly translated as capped mRNAs, and showed that Acapped CSFV IRES Fluc MS2 hairpin mRNA was unaffected by tethered MS2-4E-T, unlike the capped Fluc MS2 hairpin mRNA, which was repressed (5). HCV and CSFV are members of Flaviviruses, and their IRESes share the same host factor requirements (51). Hence 4E-T repression of bound mRNA normally requires eIF4F (except for eIF4E, see above)/eIF4B, eIF1 or eIF1A.

### Depleting 4E-T increases cellular mRNA translation and reduces silencing of microRNA-target mRNAs

**Figure 9.**
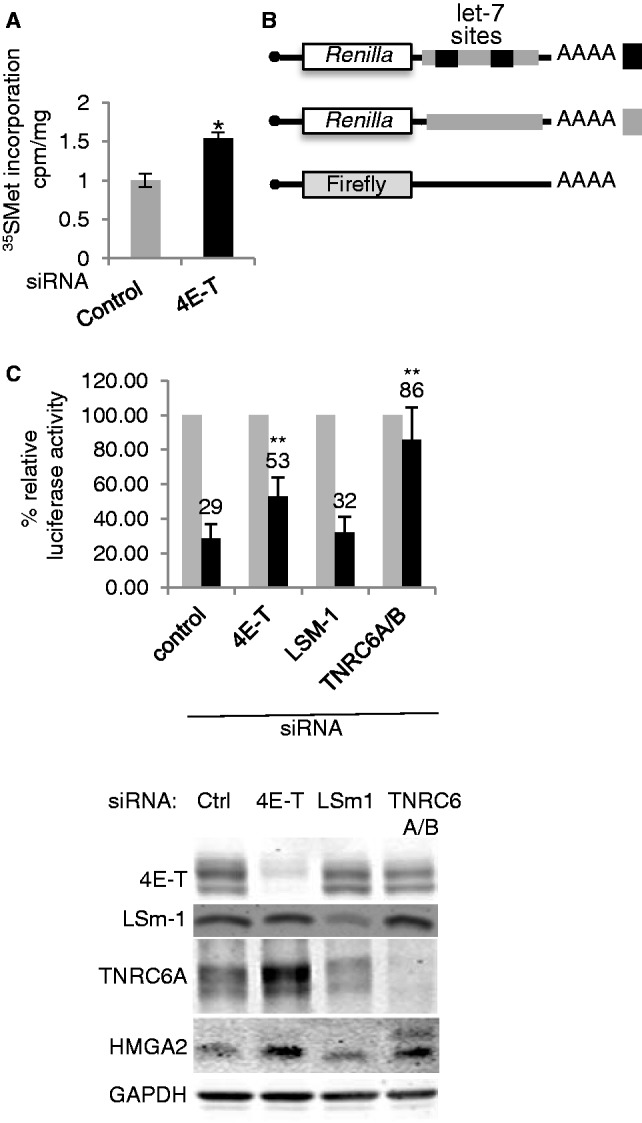
4E-T silencing enhances protein synthesis, including that of miRNA-regulated reporter and cellular mRNAs. (**A**) 35S methionine/cysteine incorporation. HeLa cells transfected with a nontargeting scrambled siRNA control and 4E-T siRNA were labelled with 35S methionine/cysteine and the amount of radiolabelled protein was quantitated by trichloroacetic acid precipitation. *P < 0.05. (**B**) 4E-T participates in miRNA-mediated gene silencing. HeLa cells were transfected with control and siRNA against 4E-T, LSm1 and TNRC6A/B. Renilla luciferase reporter mRNA with 0 or 2 let-7 miRNA target sites was transfected alongside firefly luciferase control reporter mRNA 2 days after siRNA knock-down, and luciferase activities were determined. **P < 0.01. (**C**) siRNA depletion was assessed by western blotting using indicated antibodies with GAPDH as loading control.

To examine to what extent endogenous 4E-T regulates protein synthesis, we compared the rate of synthesis with ^35^SMet labelling in HeLa cells treated with control siRNAs, and with 4E-T siRNA. Relative to control siRNA-treated cells, depletion of 4E-T increases global protein synthesis by ∼55% (Figure 9A), in line with our previous data showing that overexpression of 4E-T reduces general translation (Figure 4, Supplementary Figure S5 and Supplementary Figure S6).

Next, using *Renilla* luciferase reporters lacking or containing two let-7 microRNA binding sites in their 3′ UTR (37), we asked whether 4E-T influenced microRNA-mediated silencing. The let-7 reporter was silenced ∼4-fold in control siRNA cells, and in cells depleted of LSm1, which is not involved in this process. In contrast, depletion of 4E-T had a modest but significant effect indicating that microRNA-mediated repression is partially relieved. The same outcome was observed in the case of a second 4E-T siRNA (Supplementary Figure S9). The result with LSm1, a P-body component required for P-body assembly (20), shows that derepression on 4E-T depletion is not simply due to the loss of P-bodies. The reporter mRNAs were verified with TNRC6A/B siRNA, which almost completely relieved repression by the endogenous let-7 microRNAs (Figure 9B).

To validate the reporter assay, 4E-T and control siRNA-treated cells were assessed for extent of depletion by western blotting. In each case, the stated siRNA had the predicted effect of depleting the levels of the cognate protein (Figure 9C). We noted an up-regulation of TNRC6A levels in 4E-T depleted cells (Figure 9C); previously observed in cells depleted of eIF4A2, a critical factor in microRNA-mediated silencing (42); see ‘Discussion’ section). Importantly, 4E-T siRNA enhanced the levels of HMGA2, an endogenous target of let-7 (52), in line with the reporter data (Figure 9C and B). Altogether we concluded that 4E-T regulates the translation of cellular mRNAs, including microRNA-target mRNAs.

## DISCUSSION

### 4E-T localization in P-bodies

We demonstrate that the C-terminal region downstream of residue 694 is critical for 4E-T localization to P-bodies. Indeed, 4E-T is one of several proteins, including rck/p54, that are required for P-body assembly (23,24,53), indicating the importance of multiple interactions.

To identify the region that targets 4E-T to P-bodies, we reasoned that this would likely be common to previously characterized 4E-T homologues with similar roles in eIF4E binding, translational control of specific mRNAs and enrichment in mRNP. However we found very limited sequence homology between vertebrate 4E-T and Cup, and refocused our attention on *Drosophila* CG32016/4E-T, considerably closer to h4E-T than Cup. This sequence analysis allowed us to truncate human 4E-T from both the C-terminus, and N-terminus, removing blocks of conserved residues. Ultimately we concluded that conserved sequences in the C-terminus were critical for P-body localization. Entirely in line with this data, we observed that *Drosophila* Cup, which lacks these sequences, does not localize to P-bodies in HeLa cells, in contrast to *Drosophila* CG32016/4E-T. Interestingly, we noted that all *Drosophila* possess both Cup and 4E-T paralogues (Supplementary Figure S10), while other metazoa are restricted to a single 4E-T gene. *Drosophila* 4E-T is moderately expressed in various larval and adult tissues, including the central nervous system, with the highest levels seen in the ovary, while *Drosophila* Cup is highly expressed in both the ovary and testis (Flybase), indicating that the two paralogues may coexist, possibly to carry out distinct functions. The nematode 4E-T homologue Spn-2 gains its name from being spindle orientation defective. A mutant allele defective in normal spindle orientation in early embryos introduces a stop codon in place of Q at amino acid residue 540 (10), corresponding to residue 685 in the human protein (Supplementary Figure S1C), the truncation we defined as dominant negative for P-bodies.

4E-T is phosphorylated during meiotic maturation of *Xenopus* and mouse oocytes (5,54). A recent study reported that during oxidative stress, JNK kinase relocalizes to P-bodies and modifies several centrally located proline-directed S/T residues in h4E-T, which promote the aggregation of previously assembled P-bodies into larger granules (55). Thus as for other P-body components including Dcp1 (56) and Pat1p (57), signal transduction pathways can regulate 4E-T distribution and P-body formation.

### Effects of 4E-T on translation and mRNA decay

We observed, in reporter assays and in ^35^SMet incorporation assays that overexpression of 4E-T, but not 4E-T-Y30A nor 4E-T-YLL → AAA, downregulates general protein synthesis in HEK293 and in HeLa cells in a dose-dependent manner, in line with previous reports (see ‘Introduction’ section). We reasoned that this outcome was due to the sequestration of eIF4E by wild-type 4E-T, preventing translation initiation. Interestingly, RNAi knock-down of eIF4E does not affect global protein synthesis significantly, but rather, in a homeostatic mechanism, leads to the degradation of hypophosphorylated 4E-BP1 allowing any residual cap-binding protein to perform its function (58). In contrast, in a tether function assay, 4E-T repressed bound mRNAs in an eIF4E (or more accurately YX_4_Lϕ)-independent manner. To our knowledge, this is the first report of a protein that can affect expression of both control and bound mRNAs, via distinct mechanisms.

To date, only the canonical cap-binding protein eIF4E1 has been studied extensively in human and murine cell lines, though mammals possess additional homologues, eIF4E2 and eIF4E3, with varying capacities to bind the cap, eIF4G and 4E-BP to those of eIF4E1 (59). Potentially, these variants could interact with 4E-T at a distinct site to eIF4E1, and be responsible for YX_4_Lϕ-independent repression. However, we have recently shown that while human 4E-T indeed binds eIF4E2, as well as eIF4E1, mutagenesis of the critical tyrosine at position 30 to alanine, either alone or in combination with similar changes to the two leucine residues, fully prevented both interactions (43). There is no verified eIF4E3 antibody available as yet to check this interaction, though in any case, the levels of eIF4E3 in genome-wide proteomic analyses of mammalian cell lines are low or undetectable [summarized in (43)].

The IRES tether function experiment suggested that 4E-T repression of bound mRNA normally requires eIF4F (except for eIF4E)/eIF4B, eIF1 or eIF1A. 4E-T had no significant effects on the stability or polyadenylation of bound mRNA. However, when binding to eIF4E was prevented, modest effects on total levels were observed, involving enhanced binding to the CNOT deadenylase, followed, very likely, by enhanced decapping resulting from reduced protection of the cap structure by mutant tethered 4E-T.

Interesting parallels and contrasts can be drawn between human 4E-T and *Drosophila* Cup. *Drosophila* Cup possesses two binding sites for eIF4E, one being a canonical sequence, and the downstream one being ELEGRLR, but unlike the case with 4E-T, mutagenesis of the canonical site does not fully prevent interaction with eIF4E, while mutagenesis of the second site only partially prevents binding (8,19). Tethered *Drosophila* Cup was recently demonstrated to translationally repress bound mRNA, with the two binding sites playing different roles. The Cup protein mutant in the first (canonical) site represses translation, in the absence of mRNA decay, but promotes deadenylation, as does wild-type Cup. On the other hand, mutagenesis of the second site resulted in the large-scale decay of bound mRNA, due to decapping in addition to deadenylation (19). In contrast, 4E-T represses translation of tethered mRNA, when bound to eIF4E, with no obvious effects on mRNA levels or processing, and eIF4E binding reduces the interaction with CNOT deadenylase subunits. In *C. elegans* embryos lacking functional IFET-1 (Spn-2), mRNAs undergo partial poly(A) shortening rather than elongation (12), also implying that normally 4E-T does not promote deadenylation.

How 4E-T (and Cup) represses translation when bound to mRNA normally, presumably via 3′ UTR-binding proteins (rather than a tethering protein) remains to be investigated. Our results indicate that these putative RNA-binding proteins do not require the involvement of P-bodies, as repression by the tethered protein occurs in their absence, though we cannot exclude the participation of individual factors dispersed in the cytoplasm. In agreement, Cup lacks P-body localization sequences, and depletion of several P-body components from S2 cells did not relieve bound mRNA repression (19). Moreover, the repression mechanism *per se* also does not appear to involve the conserved central CHD region, in either Cup (19) or 4E-T (this work). With regard to eIF4E, while our and others data clearly demonstrate the apparent complete loss of binding of the cap-binding protein on mutagenesis of the YX_4_Lϕ motif in 4E-T, we cannot exclude the possibility that such a mutant 4E-T represses bound mRNA due to its being tethered and binding—albeit very weakly—eIF4E and hence the cap, occluding ribosome recruitment.

### Possible targets of 4E-T

In line with our overexpression data, depletion of 4E-T resulted in enhanced rates of global protein synthesis. Levels of 4E-T, compared with eIF4E, 4E-BP and eIF4G in mammalian cells are relatively low ((43) and refs therein), so this was not entirely anticipated. The increased translation rate may simply reflect a consistent increase in access to eIF4E for all mRNAs, or may also indicate enhancement of specific subsets of mRNAs. Previously it was shown that depletion of 4E-T stabilizes reporter and endogenous ARE mRNAs (21), and we demonstrate here that depletion of 4E-T results in the partial relief of microRNA-mediated silencing, of both reporter and endogenous mRNAs. Similarly, depletion of *Drosophila* CG32016/4E-T results in modest relief of a Vha68–1 reporter mRNA with two mir-9b binding sites in S2 cells (60). MicroRNAs silence their target mRNAs in a two-step mechanism whose primary site of action is translational repression, and which results in mRNA decay initiated by deadenylation followed by decapping (42,61–63). Our previous study showed that silencing of eIF4A2 had a more significant effect on miRNA reporter mRNA silencing (42) than we report here for 4E-T. Though the detailed mechanism of eIF4A2- and 4E-T-mediated microRNA silencing remains to be elucidated, it is interesting to note that both can bind CNOT deadenylase, suggesting that this may be their primary site of action. Interestingly too, silencing of both 4E-T and eIF4A2 results in enhanced levels of GW182/TNRC6A, which is not seen in cells depleted of other eIF4F translation initiation factors or of LSm1 [Figure 9; (42)]. However, this increased GW182 level is unlikely to be functional, and is not responsible for the observed relief of silencing with eIF4A2 siRNA (indirectly perhaps resulting from sequestration of other miRISC components), as this was unaffected when combined with a low concentration of GW182 siRNA, sufficient to reduce GW182 levels to those in untreated cells (unpublished observations). More plausibly, the increase in GW182 in cells depleted of 4E-T or eIF4A2 may indicate that all three are involved in the miRNA pathway. Together these observations can be reconciled by proposing that the initial role of 4E-T is to repress the translation of subsets of mRNAs, which may result in their degradation.

Lastly, we consider the question of the possible significance of 4E-T residence in P-bodies. We have shown that a delocalized mutant protein, even one that disrupts endogenous P-bodies, repressed tethered mRNAs just as well as the localized protein, indicating that repression *per se* does not involve P-bodies. Similarly, *C. elegans* Spn-2 bridges eIF4E and OMA-1, an RNA-binding protein, and the *mei-1* (meiotic katanin) 3′ UTR to repress its translation but this does not require P-granules as it is still observed in *pgl-1* mutants (10). Perhaps, we speculate, 4E-T recruits a subset of mRNAs to these RNP granules?

Our work has demonstrated the conserved yet unpredicted and surprising translational control of bound mRNAs by 4E-T, which does not involve eIF4E, nor P-body components. Future investigations will focus on identifying the protein(s), presumably RNA-binding proteins that naturally tether 4E-T to mRNA, and how they occlude ribosome binding. 4E-T and the related Cup proteins are particularly highly expressed in oocytes and ovaries in flies, worms and *Xenopus*, and exert tight control over maternal mRNA translation. Of note, Kasippillai *et al.* (64) recently reported that mutations in h4E-T at Ser 496 to a stop codon are associated with primary ovarian insufficiency (premature ovarian failure). It will clearly be important thus to determine which mRNAs are regulated by 4E-T proteins in early development and in the soma.

## SUPPLEMENTARY DATA

Supplementary Data are available at NAR Online, including [44].

## FUNDING

Wellcome Trust [084885/Z/08/Z] and BBSRC [BB/J00779X/1 to N.S.]; MRC [G0902052 to M.B.]; Gates Cambridge Scholarship postgraduate funding (to A.K.). Funding for open access charge: Wellcome Trust and BBSRC.

*Conflict of interest statement*. None declared.

## Supplementary Material

Supplementary Data

## References

[1] Furic L, Rong L, Larsson O, Koumakpayi I, Yoshida K, Brueschke A, Petroulakis E, Robichaud N, Pollak M, Gaboury LA (2010). eIF4E phosphorylation promotes tumorigenesis and is associated with prostate cancer progression. Proc. Natl Acad. Sci. USA.

[2] Rhoads R (2009). eIF4E: new family members, new binding partners, new roles. J. Biol. Chem..

[3] Jackson RJ, Hellen C, Pestova T (2010). The mechanism of eukaryotic translation initiation and principles of its regulation. Nat. Rev. Mol. Cell. Biol..

[4] Kinkelin K, Veith K, Grünwald M, Bono F (2012). Crystal structure of a minimal eIF4E-Cup complex reveals a general mechanism of eIF4E regulation in translational repression. RNA.

[5] Minshall N, Reiter M-H, Weil D, Standart N (2007). CPEB interacts with an ovary-specific eIF4E and 4E-T in early Xenopus oocytes. J. Biol. Chem..

[6] Fernández-Miranda G, Méndez R (2012). The CPEB-family of proteins, translational control in senescence and cancer. Ageing Res. Rev..

[7] Wilhelm JE, Hilton M, Amos Q, Henzel W (2003). Cup is an eIF4E binding protein required for both the translational repression of oskar and the recruitment of Barentsz. J. Cell Biol..

[8] Nelson MR, Leidal AM, Smibert CA (2004). *Drosophila* Cup is an eIF4E-binding protein that functions in Smaug-mediated translational repression. EMBO J..

[9] Nakamura A, Sato K, Hanyu-Nakamura K (2004). *Drosophila* Cup is an eIF4E binding protein that associates with Bruno and regulates oskar mRNA translation in oogenesis. Dev. Cell.

[10] Li W, DeBella LR, Guven-Ozkan T, Lin R, Rose LS (2009). An eIF4E-binding protein regulates katanin protein levels in *C. Elegans* embryos. J. Cell Biol..

[11] Guven-Ozkan T, Robertson SM, Nishi Y, Lin R (2010). zif-1 translational repression defines a second, mutually exclusive OMA function in germline transcriptional repression. Development.

[12] Sengupta MS, Low WY, Patterson JR, Kim HM, Traven A, Beilharz TH, Colaiácovo MP, Schisa JA, Boag PR (2013). ifet-1 is a broad-scale translational repressor required for normal P granule formation in C. elegans. J. Cell Sci..

[13] Keyes LN, Spradling AC (1997). The *Drosophila* gene fs(2)cup interacts with otu to define a cytoplasmic pathway required for the structure and function of germ-line chromosomes. Development.

[14] Chekulaeva M, Hentze MW, Ephrussi A (2006). Bruno acts as a dual repressor of oskar translation, promoting mRNA oligomerization and formation of silencing particles. Cell.

[15] Jeske M, Moritz B, Anders A, Wahle E (2011). Smaug assembles an ATP-dependent stable complex repressing nanos mRNA translation at multiple levels. EMBO J..

[16] Schisa JA (2012). New insights into the regulation of RNP granule assembly in oocytes. Int. Rev. Cell Mol. Biol..

[17] SenGupta DJ, Zhang B, Karemer B, Pochart P, Fields S, Wickens M (1996). A three-hybrid system to detect RNA-protein interactions *in vivo*. Proc. Natl Acad. Sci. USA.

[18] Dostie J, Ferraiuolo M, Pause A, Adam SA, Sonenberg N (2000). A novel shuttling protein, 4E-T, mediates the nuclear import of the mRNA 5' cap-binding protein, eIF4E. EMBO J..

[19] Igreja C, Izaurralde E (2011). CUP promotes deadenylation and inhibits decapping of mRNA targets. Genes Dev..

[20] Andrei MA, Ingelfinger D, Heintzmann R, Achsel T, Rivera-Pomar R, Lührmann R (2005). A role for eIF4E and eIF4E-transporter in targeting mRNPs to mammalian processing bodies. RNA.

[21] Ferraiuolo MA, Basak S, Dostie J, Murray EL, Schoenberg DR, Sonenberg N (2005). A role for the eIF4E-binding protein in P-body formation and mRNA decay. J. Cell Biol..

[22] Lee HC, Cho H, Kim YK (2008). Ectopic expression of eIF4E-transporter triggers the movement of eIF4E into P-bodies, inhibiting steady-state translation but not the pioneer round of translation. Biochem. Biophys. Res. Commun..

[23] Eulalio A, Behm-Ansmant I, Izaurralde E (2007). P bodies: at the crossroads of post-transcriptional pathways. Nat. Rev. Mol. Cell Biol..

[24] Parker R, Sheth U (2007). P bodies and the control of mRNA translation and degradation. Mol. Cell.

[25] Kulkarni M, Ozgur S, Stoecklin G (2010). On track with P-bodies. Biochem. Soc. Trans..

[26] Standart N, Minshall N (2008). Translational control in early development: CPEB, P-bodies and germinal granules. Biochem. Soc. Trans..

[27] Decker CJ, Teixeira D, Parker R (2007). Edc3p and a glutamine/asparagine-rich domain of Lsm4p function in processing body assembly in *Saccharomyces cerevisiae*. J. Cell Biol..

[28] Eulalio A, Behm-Ansmant I, Schweizer D, Izaurralde E (2007). P-body formation is a consequence, not the cause of RNA-mediated gene silencing. Mol. Cell. Biol..

[29] Stalder L, Muhlemann O (2009). Processing bodies are not required for mammalian nonsense-mediated mRNA decay. RNA.

[30] Franks TM, Lykke-Andersen J (2007). TTP and BRF proteins nucleate processing body formation to silence mRNAs with AU-rich elements. Genes Dev..

[31] Takahashi S, Sakurai K, Ebihara A, Kajiho H, Saito K, Kontani K, Nishina H, Katada T (2011). RhoA activation participates in rearrangement of processing bodies and release of nucleated AU-rich mRNAs. Nucleic Acids Res..

[32] Glasmacher E, Hoefig KP, Vogel KU, Rath N, Du L, Wolf C, Kremmer E, Wang X, Heissmeyer V (2010). Roquin binds inducible costimulator mRNA and effectors of mRNA decay to induce microRNA-independent post-transcriptional repression. Nat. Immunol..

[33] Lee EK, Kim HH, Kuwano Y, Abdelmohsen K, Srikantan S, Subaran SS, Gleichmann M, Mughal MR, Martindale JL, Yang X (2010). hnRNP C promotes APP translation by competing with FMRP for APP mRNA recruitment to P bodies. Nat. Struct. Mol. Biol..

[34] Carbonaro M, O'Brate A, Giannakakou P (2011). Microtubule disruption targets HIF-1alpha mRNA to cytoplasmic P-bodies for translational repression. J. Cell Biol..

[35] Cui YH, Xiao L, Rao JN, Zou T, Liu L, Chen Y, Turner DJ, Gorospe M, Wang JY (2012). miR-503 represses CUG-binding protein 1 translation by recruiting CUGBP1 mRNA to processing bodies. Mol. Biol. Cell.

[36] Pillai RS, Bhattacharyya SN, Artus CG, Zoller T, Cougot N, Basyuk E, Bertrand E, Filipowicz W (2005). Inhibition of translational initiation by let-7 microRNA in human cells. Science.

[37] Kong YW, Cannell IG, de Moor CH, Hil lK, Garside PG, Hamilton TL, Meijer HA, Dobbyn HC, Stoneley M, Spriggs KA (2008). The mechanism of micro-RNA-mediated translation repression is determined by the promoter of the target gene. Proc. Natl Acad. Sci. USA.

[38] Winkler GS, Mulder KW, Bardwell VJ, Kalkhoven E, Timmers HT (2006). Human Ccr4-Not complex is a ligand-dependent repressor of nuclear receptor-mediated transcription. EMBO J..

[39] Aslam A, Mittal S, Koch F, Andrau JC, Winkler GS (2009). The Ccr4-NOT deadenylase subunits CNOT7 and CNOT8 have overlapping roles and modulate cell proliferation. Mol. Biol. Cell.

[40] Marnef A, Maldonado M, Bugaut A, Balasubramanian S, Kress M, Weil D, Standart N (2010). Distinct functions of maternal and somatic Pat1 protein paralogs. RNA.

[41] Minshall N, Allison R, Marnef A, Wilczynska A, Standart N (2010). Translational control assessed using the tethered function assay in Xenopus oocytes. Methods.

[42] Meijer HA, Kong YW, Lu WT, Wilczynska A, Spriggs RV, Robinson SW, Godfrey JD, Willis AE, Bushell M (2013). Translational repression and eIF4A2 activity are critical for microRNA-mediated gene regulation. Science.

[43] Kubacka D, Kamenska A, Broomhead H, Minshall N, Darzynkiewicz E, Standart N (2013). Investigating the consequences of eIF4E2 (4EHP) interaction with on its cellular distribution in HeLa cells. PLos One.

[44] Dereeper A, Guignon V, Blanc G, Audic S, Buffet S, Chevenet F, Dufayard JF, Guindon S, Lefort V, Lescot M (2008). Phylogeny.fr: robust phylogenetic analysis for the non-specialist. Nucleic Acids Res..

[45] Braun JE, Tritschler F, Haas G, Igreja C, Truffault V, Weichenrieder O, Izaurralde E (2010). The C-terminal alpha-alpha superhelix of Pat is required for mRNA decapping in metazoa. EMBO J..

[46] Stade K, Ford CS, Guthrie C, Weis K (1997). Exportin 1 (Crm1p) is an essential nuclear export factor. Cell.

[47] Ozgur S, Chekulaeva M, Stoecklin G (2010). Human Pat1b connects deadenylation with mRNA decapping and controls the assembly of Processing-bodies. Mol. Cell. Biol..

[48] Totaro A, Renzi F, La Fata G, Mattioli C, Raabe M, Urlaub H, Achsel T (2011). The human Pat1b protein: a novel mRNA deadenylation factor identified by a new immunoprecipitation technique. Nucleic Acids Res..

[49] Schoenberg DR, Maquat LE (2012). Regulation of cytoplasmic mRNA decay. Nat. Rev. Genet..

[50] Wahle E, Winkler GS (2013). RNA decay machines: deadenylation by the Ccr4-not and Pan2-Pan3 complexes. Biochim. Biophys. Acta..

[51] Pestova TV, Shatsky IN, Fletcher SP, Jackson RJ, Hellen CU (1998). A prokaryotic-like mode of cytoplasmic eukaryotic ribosome binding to the initiation codon during internal translation initiation of hepatitis C and classical swine fever virus RNAs. Genes Dev..

[52] Mayr C, Hemann MT, Bartel DP (2007). Disrupting the pairing between let-7 and Hmga2 enhances oncogenic transformation. Science.

[53] Serman A, Le Roy F, Aigueperse C, Kress M, Dautry F, Weil D (2007). GW body disassembly triggered by siRNAs independently of their silencing activity. Nucleic Acids Res..

[54] Villaescusa JC, Allard P, Carminati E, Kontogiannea M, Talarico D, Blasi F, Farookhi R, Verrotti AC (2006). Clast4, the murine homologue of human eIF4E-Transporter, is highly expressed in developing oocytes and post-translationally modified at meiotic maturation. Gene.

[55] Cargnello M, Tcherkezian J, Dorn JF, Huttlin EL, Maddox PS, Gygi SP, Roux PP (2012). 4E-T phosphorylation by JNK promotes stress-dependent P-body assembly. Mol. Cell. Biol..

[56] Rzeczkowski K, Beuerlein K, Müller H, Dittrich-Breiholz O, Schneider H, Kettner-Buhrow D, Holtmann H, Kracht M (2011). c-Jun N-terminal kinase phosphorylates DCP1a to control formation of P bodies. J. Cell Biol..

[57] Ramachandran V, Shah KH, Herman PK (2011). The cAMP-dependent protein kinase signaling pathway is a key regulator of P body foci formation. Mol. Cell.

[58] Yanagiya A, Suyama E, Adachi H, Svitkin YV, Aza-Blanc P, Imataka H, Mikami S, Martineau Y, Ronai ZA, Sonenberg N (2012). Translational homeostasis via the mRNA cap-binding protein, eIF4E. Mol. Cell.

[59] Joshi B, Lee K, Maeder DL, Jagus R (2005). Phylogenetic analysis of eIF4E-family members. BMC Evol. Biol..

[60] Rehwinkel J, Behm-Ansmant I, Gatfield D, Izaurralde E (2005). A crucial role for GW182 and the DCP1:DCP2 decapping complex in miRNA-mediated gene silencing. RNA.

[61] Bazzini AA, Lee MT, Giraldez AJ (2012). Ribosome profiling shows that miR-430 reduces translation before causing mRNA decay in zebrafish. Science.

[62] Djuranovic S, Nahvi A, Green R (2012). miRNA-mediated gene silencing by translational repression followed by mRNA deadenylation and decay. Science.

[63] Béthune J, Artus-Revel CG, Filipowicz W (2012). Kinetic analysis reveals successive steps leading to miRNA-mediated silencing in mammalian cells. EMBO Rep..

[64] Kasippillai T, Macarthur DG, Kirby A, Thomas B, Lambalk CB, Daly MJ, Welt CK (2013). Mutations in eIF4ENIF1 Are Associated With Primary Ovarian Insufficiency. J. Clin. Endocrinol. Metab..

