# Supplementary material for: Human 4E-T represses translation of bound mRNAs and enhances microRNA-mediated silencing
Source: Nucleic Acids Res. 2013 Dec 13;42(5):3298–313. doi: 10.1093/nar/gkt1265 (PMC3950672; doi:10.1093/nar/gkt1265)
Supplement: Supplementary Data [file supp_42_5_3298__index.html]

Human 4E-T represses translation of bound mRNAs and enhances microRNA-mediated silencing — Human 4E-T represses translation of bound mRNAs and enhances microRNA-mediated silencing — Supplementary Data 

# Human 4E-T represses translation of bound mRNAs and enhances microRNA-mediated silencing

## Supplementary Data

files

**Files in this Data Supplement:**

- Supplementary Data - pdf file
