# Supplementary material for: Human 4E-T represses translation of bound mRNAs and enhances microRNA-mediated silencing
Source: Nucleic Acids Res. 2013 Dec 13;42(5):3298–313. doi: 10.1093/nar/gkt1265 (PMC3950672; doi:10.1093/nar/gkt1265)
Supplement: Supplementary Data [file supp_gkt1265_nar-02620-v-2013-File005.pdf]

Supplementary Material

Figures Legends

Suppl Fig. 1

Sequence alignment using ClustalW2 to highlight sequences conserved in 4E-T proteins and in Cup. Amino acid numbers are indicated, conserved regions are in red. **A.** The N-terminal regions of human, mouse, *Xenopus*, *C. elegans* and *Drosophila* 4E-T (CG32016) proteins, and Cup. **B**. The CHD domain. **C.** Clustal analysis of the C-terminal regions of human, mouse, *Xenopus* and *Drosophila* 4E-T (CG32016) proteins. Arrows indicate location of truncations in the identification of P-body localisation experiments. Below are shown the equivalent regions of *Ce* 4E-T (Spn-2), which shares some of the conserved sequences identified in **C**, and of Cup, which does not show homology in this region.

Suppl. Fig. 2

h4E-T and DmCG32016 are nucleocytoplasmic shuttling proteins, unlike Cup. HeLa cells transfected with GFP-4E-T/Cup fusion proteins as shown were treated with LMB for 5 hours prior to fixation. Cells were also immunostained with p54 antibodies to mark P-bodies. Scale 10 μm.

Suppl. Fig. 3

Confocal microscopy of untransfected HeLa cells (-) and of cells transfected with indicated GFP-4E-T plasmids. Cells were stained with 4E-T, eIF4E (**A**) and p54 (**B**) antibodies. Scale 10 μm.

Suppl. Fig. 4

Repression by 4E-T requires BoxB sites in reporter mRNA. Tether function assay with NHA-GFP and NHA-4E-T, using *Renilla* luciferase mRNA with and without 3'UTR BoxB elements.

Suppl. Fig. 5

Wild-type but not the eIF4E-binding mutant of 4E-T decreases ^{35}S methionine incorporation. **A.** HEK293 cells were untransfected (-) or transfected with GFP, GFP-4E-T and GFP-4E-T-Y30A plasmids and labelled with ^{35}S methionine/cysteine for 15 min. Cells were harvested, and the amount of radiolabeled protein was quantitated by TCA precipitation. Average of 3 independent experiments. **B.** Samples were run on 15% SDS-PAGE and autoradiographed.

Shown are portions of the film with 4E-T proteins and GFP. All three are expressed at similar levels.

Suppl. Fig. 6

Tether function assay with a concentration series of indicated NHA-proteins and *Renilla* luciferase-BoxB and Firefly-luciferase control mRNAs. HEK293 cells were transfected with varying amounts of each NHA protein plasmid (indicated in ng) in 24 well plates, and fixed amounts of luciferase plasmids. (Approximately four fold more DNA is used in a typical 6 well plate transfection.) **A**. The resulting separate luciferase activities are shown, as is their relative ratio. **B**. Western blot analysis of transfected lysates developed with HA antibodies.

Suppl. Fig. 7

Tether function assay with 4E-T proteins missing the NLS or CHD sequences. Assay performed as in Fig. 4 and 5.

Suppl. Fig. 8

Tether function assay with IRES *Renilla* reporter mRNA. Separate firefly and *Renilla* luciferase activities and mRNA levels (normalised to GAPDH) are shown, as indicated.

Suppl. Fig. 9

4E-T participates in miRNA-mediated gene silencing. HeLa cells were transfected with control and two independent siRNA against 4E-T as well as TNRC6A/B as a positive control. **A**. *Renilla* luciferase reporter mRNA with 0 or 2 let-7 miRNA target sites was transfected alongside firefly luciferase control reporter mRNA 2 days after siRNA knockdown, and luciferase activities were determined. *, $P<0.05$. **B.** siRNA depletion was assessed by western blotting using indicated antibodies with vinculin as loading control.

Suppl. Fig. 10

Phylogeny tree of Cup and CG32016-like (4E-T) proteins from *Drosophila melanogaster* (NM_078769 and NM_166798 resp.), *sechellia* (XM_002035928 and XM_002044275 resp.), *grimshawi* (XM_001992943 XM_001996463 resp.), *yakuba* (GE14055 and GE14496 resp.) and *willistoni* (XM_002066395 and XM_002072529 resp.) according to phylogeny.fr (64). Branch length is proportional to the number of substitutions per site.

Suppl. Table 1

Oligonucleotide sequences used in cloning and mutagenesis

Supplementary reference

64. Dereeper, A., Guignon, V., Blanc, G., Audic, S., Buffet, S., Chevenet, F., Dufayard, J.F., Guindon, S., Lefort, V., Lescot, M. *et al*. (2008) Phylogeny.fr: robust phylogenetic analysis for the non-specialist. *Nucleic Acids Res*, **36**, W465-469.

A. The eIF4E-binding site region

```
Hs4E-T        ..PPASKCPHRYTKEELLDIKE---------LPHSKQRPSCLS-EKYDSDGVWDPEKWHASLY 71
Mm4E-T        ..LPTSKSPHRYTKEELLDIKE---------RPYSKQRPSCLS-EKYDSDGVWDPEKWHASLY 70
Xl4E-T        ..PPRGRSHHSYSKEELLDIKE---------LPHSKERPSCLL-DKYDSDGVWDPEKWHSSLY 69
Dm4E-T          MDTSKISARYSKVDLLALRY---------EGKSRQRPQCSTRLELQTLGFWKINLNTAALT 52
C.e.4E-T      ..TLPTVPFKAYTRERMMELRT---------TKASMTRPENLSEDFNGEDGKFSPLKWLEHRW 73
D.m.Cup       ..VVSSKPVKSYTRSRLMDIRNGMFNALMHRSKESFVMPRIATCDDIELEGRLRRMNIWRTSD 378
                         *::  :: ::             *   *           *
```

B The Cup Homology (CHD) region

```
Hs4E-T        ..DSKRVFGERRRNDSYTEEE--PEWFSAGPTSQSETIELTGFDDKILEEDHKGRKRT--R 254
Mm4E-T        ..DSKRVFGERRRNDSYTEEE--PEWFSAGPTSQSETIELTGFDDKILEEDHKGRKRT--R 253
Xl4E-T        ..DGKRIFGERRRNDSCTEEE--PEWFSAGPTSQSETIELIGFDDKILEEDQKGRKRT--R 253
C.e.          ..GAKFAPRREERLTSLSGSEKLPEW-ADGPTTMDDMIELRGFDEPKKVKNKKNPKEK--K 233
Dm4E-T        ..EPFSTQSRSKRGNSYLIHEE-PEWFSAGPKSQLETIDLHGFEDLEKNEERSVTEDK--N 364
D.m.Cup       ..KPKSRDVEDRHHRRYRNQNEEPEWFSCGPTSRLDTIELCGFDEDEEKMLKEGNKNHGLG 605
                       . .:       :  *** : **.:  : *:* **::      :.  :
```

Suppl. Fig. 1

C

The C-terminal region

```
                    694                    716
Hs4E-T      690.SMLSPSFTPTSVIRKMYESKEK-SKEEPASGKAALGDSKEDTQKASEENLLSSSSVPSAD
Mm4E-T      689.SMLSPSFTPTSVIRKMYESREK-TKEEMAPGMVVPGDGKEDTQKTSEENLLSSNPIPNTD
Xl4E-T      680.SMLSPSFTPTSVIRKMYESKEK-IKDEPTEKQNVSG--KEGGQGTLEDGSLPNSIMQDSP
Dm4E-T      714.SPTPLAFTPTSVLRKMTADKDTQSPSTYCQNPQYHVHQQNAKQVGTRENVLEPQLTATMA
                *  . :******:***  .::.   .            ::  *   .:. *  .

                                                     845
Hs4E-T          RDSSPTTNSKLSALQRSSCSTPLSQANRYTKEQDYRPKATGRKTPTLASPVPTTPFLRPV
Mm4E-T          QDSS-TTNPKLSTLQRSSCSTPLSQTSRYTKEQDY[illegible]TAGRKTPTLASPVPGTPFLRPT
Xl4E-T          QDTSLVMGNKVSVSRHTSCTPPLPHQSRYTKDQDY[illegible]STGRRTPTVSSPVPGASFLQPL
Dm4E-T          VQPRMILGGGNFAIGQN--NQHLSPNMSQSRNQQVI[illegible]TSGN-----MQMVHGKTFGRPI
                 :.    .    .  :.  .  *.     :::*:    ::*.      . *   .* :*

Hs4E-T          HQVPLVPHVPMVRPAHQLHPGLVQRMLAQGVHPQHLPSLLQTGVLPPGMDLSHLQGISGP
Mm4E-T          HQVPLVPHVPIVRPAHQLHPGLVQRLIAQGVHPQHLPSLLQAGVLPPGIDMAPLQGLSGP
Xl4E-T          HQLPIVP---VVRPSHQLHPGLVQRMLAQGVPPQRL-SLLQAGLLPPGIDLPPLHALSPS
Dm4E-T          LKGGLNS----MPHSNSALPFTAHKIEMQPIHQPHLQQQQHRFKAVQSVESNLNTESVHQ
                 :  : .    :  ::.  *  .:::  * :   :* .  :      .::

Hs4E-T          ILGQPFYPLP-AASHPLLNPRPGTPLHLAMVQQQLQRSVLHPPGSGSHAAAVSVQTTPQN
Mm4E-T          LLGQPLYPLVSAASHPLLNPRPGTPLHLAVMQQQLQRSVLHPPGSSSQAAAISVQT-PQN
Xl4E-T          VLGQPFYPLS-PAGHPLLNQRPANPLQLAMMQQSMQRS-----GSGLHAP-------PLG
Dm4E-T          NITSPVGWHQLYMQHQQQHHHTRQQLSQRVIYGEMHRQ-----SNPQMSPPVPGFSDSSD
                 : .*.        *   : :.   *   ::  .::*.     ..   :.       . .

Hs4E-T          VPSRSGLPHMHSQLEHRPSQRSSSPVGLAKWFGSDVLQQP----LPSMPA-KVISVDELE
Mm4E-T          VPSRSGMPHMHSQLEHRTSQRSSSPVGLAKWFGSDVLQQP----LPSMPT-KVISVDELE
Xl4E-T          MTNR---PNMY--MEQRASQRSSSPVGLAKWFGSDVLQQP----LPSMPA-KVISVDELE
Dm4E-T          SGNVIKANSLTSPSYQRDERISSPTNQLAQWFSPELLAKASAGKLPLLNVNQALSLEEFE
                  .      :     :* .: **..  **:**..::* :.    ** : . :.:*::*:*

Hs4E-T          YRQ 985
Mm4E-T          YRQ 983
Xl4E-T          YRQ 953
Dm4E-T          RSIQHSSGVVHN 1010
```

```
>Ce4E-T
541.HHQHKGRTPAMIPASVQRQLQKSSSNADQKKEKTSQSPPESNQETSDAHNQS
DAMNQLKKLHMQQNYANMVQAMNSGVGWARGNGVVNGQQQHPQQQLPPNVQMLMAQ
HQQAQMQHLKMMMSRAQQQHMLMAKIAQMQQQQAQMANMQERQGPSHNQQQHQPVV
PSELSQVGPIQTPLEKLLASVGVQGSQFTGSGDRIPSSVRPMSLEDLEKQLTAVPK 761

>DmCup
715.MSFFANEGNSSSSSLNEFFKQAINQGHGNNPEQPKSLGHIGQMPSVDQLEAK
WRRNSLNNVGETANKQTDNFQKLIGSLSSAKPQSQAVGYDAISNFIMQQQQYQQQQ
QKQHLIIQQQQQHTAFLASLQLKAILGRADTQLLLLRLTKGEISKHGLLVQLANPR
LTDMDREAITAVLQFTNTQQQQQQHKQQLDMLSSTVIASQLQNLHNLAIVQQTLAA
RQQPQHNPQTQAPHQLSQEDLQAHANVIMRNAVMKRKIEEQTSKLINGGAKHQAQQ
QYLNRGQQRQARPDANSNALLHALISGGGNNHASGYPMNGQPQKHHSNLRFGDNQN
FQSFESNQPHFATQYKQQYQQSQQQHPHQQPQQLNSLHQNNAGAVNSFNKAQMQAQ
SAISMLPNSGDEFH 1117
```

Suppl. Fig. 1

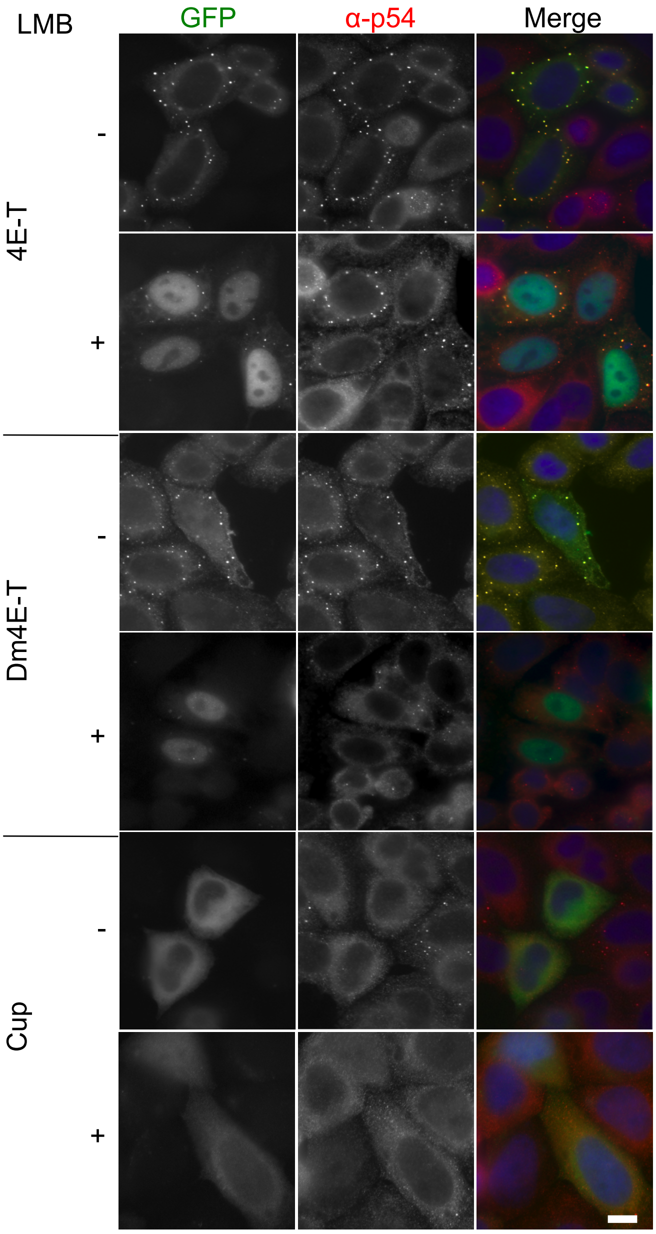

Suppl. Fig. 2

A

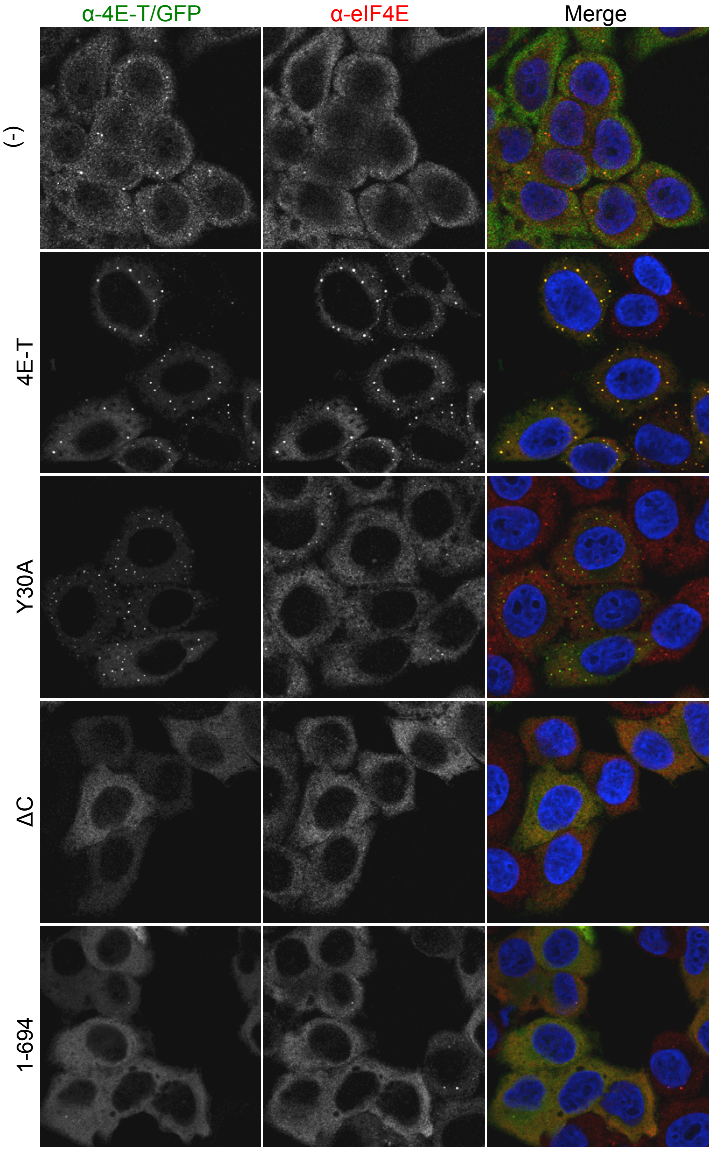

Suppl. Fig. 3

B

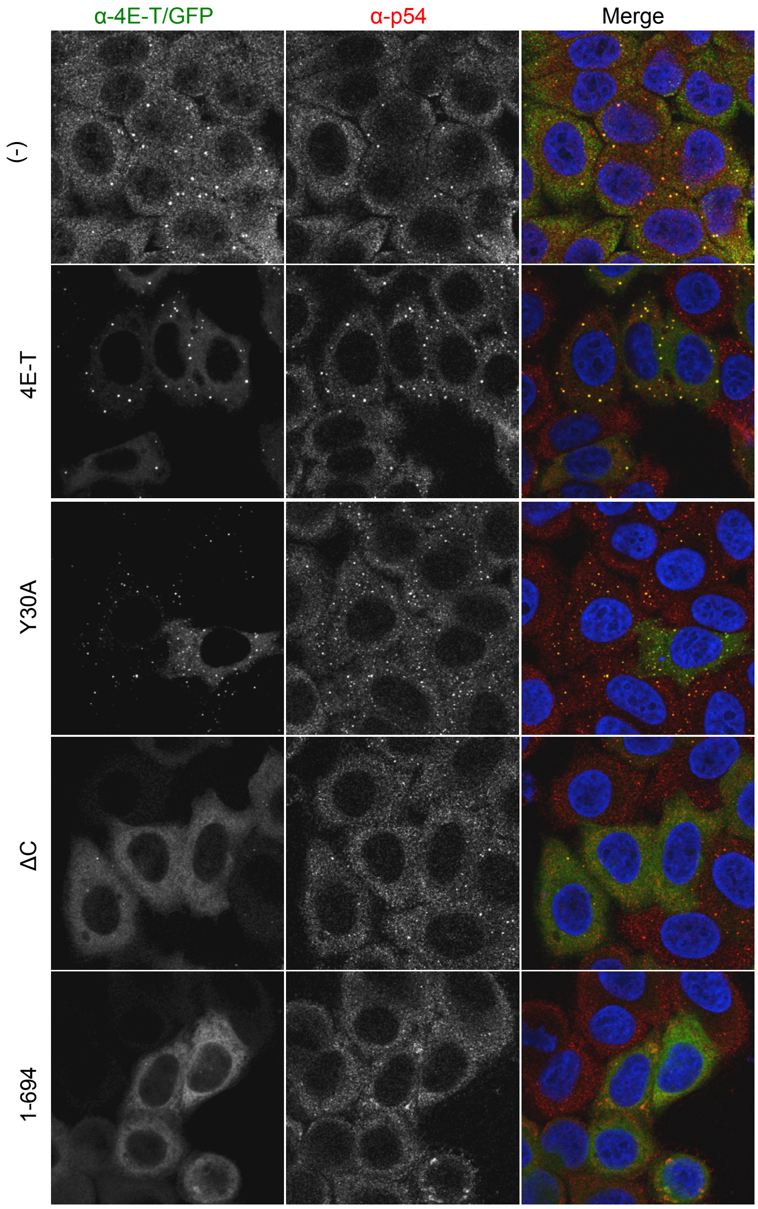

Suppl. Fig. 3

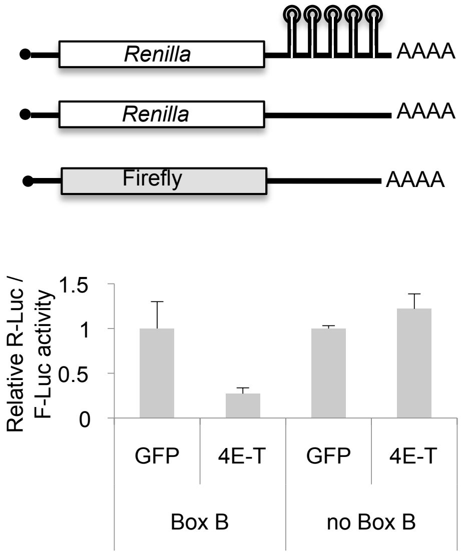

Suppl. Fig. 4

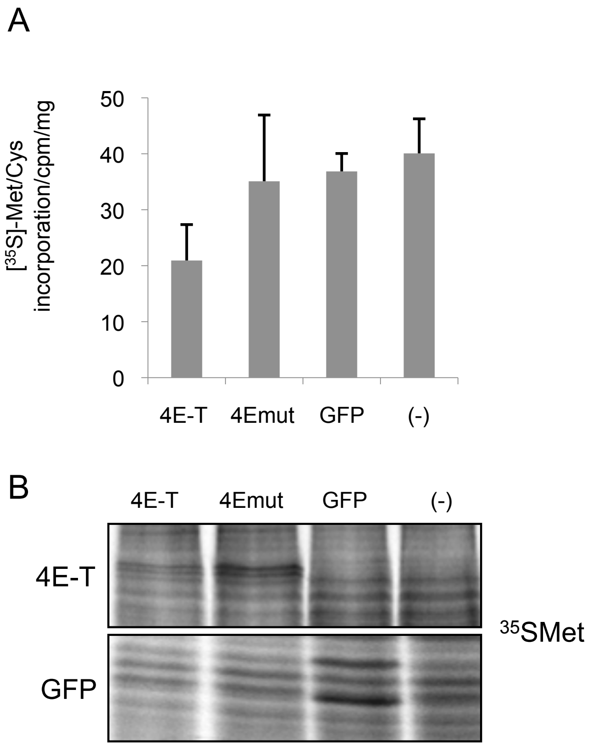

Suppl. Fig. 5

A

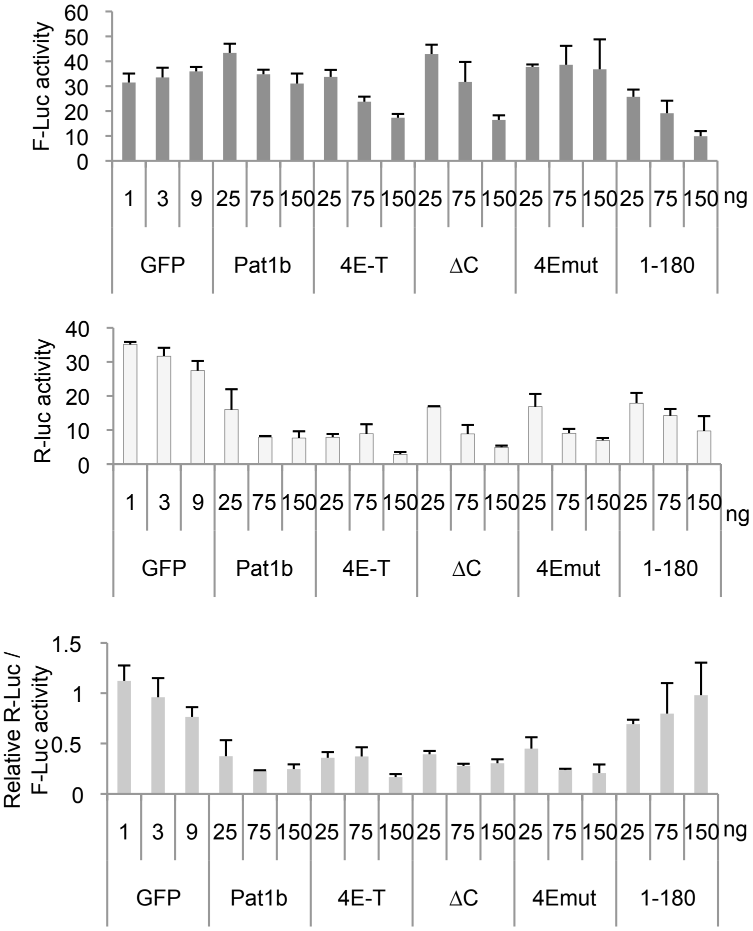

B

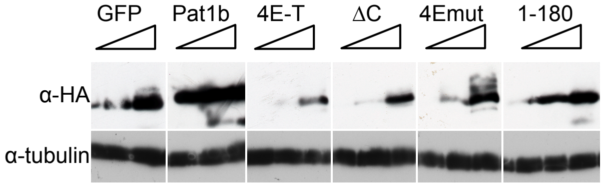

Suppl. Fig. 6

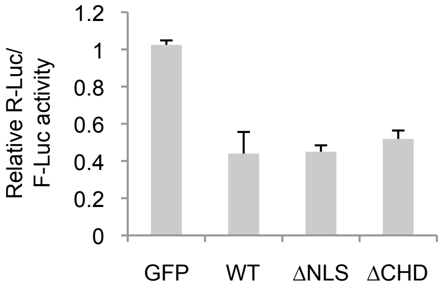

Suppl. Fig. 7

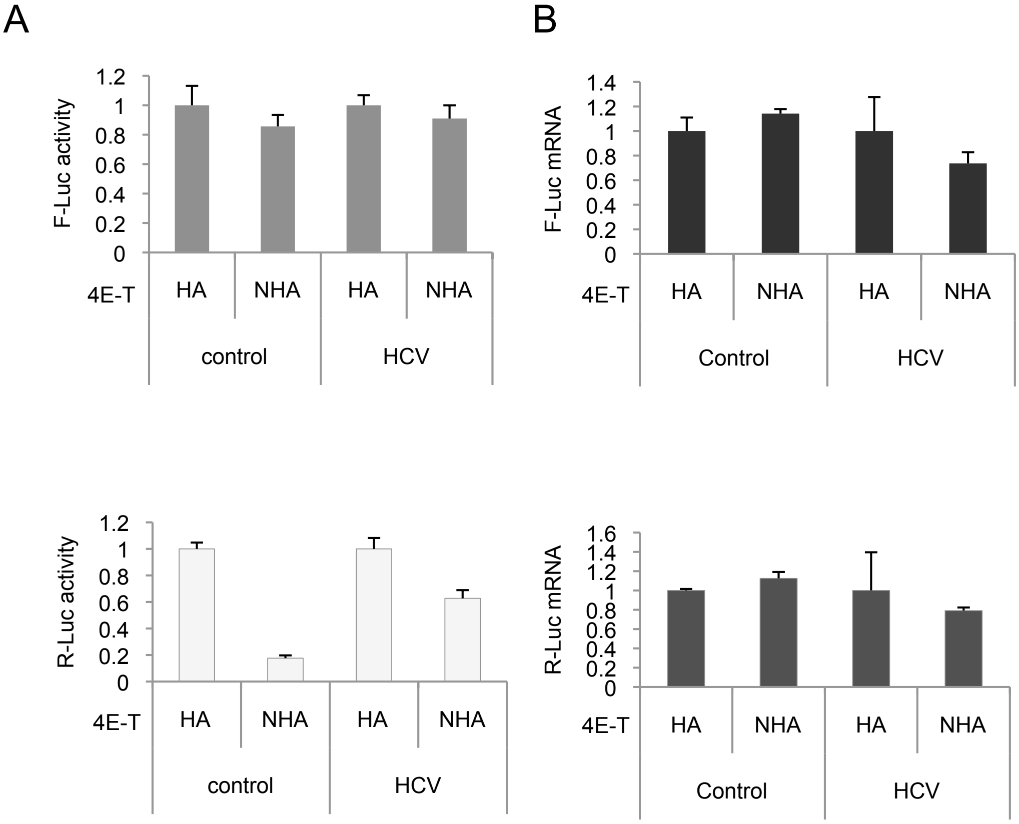

Suppl. Fig. 8

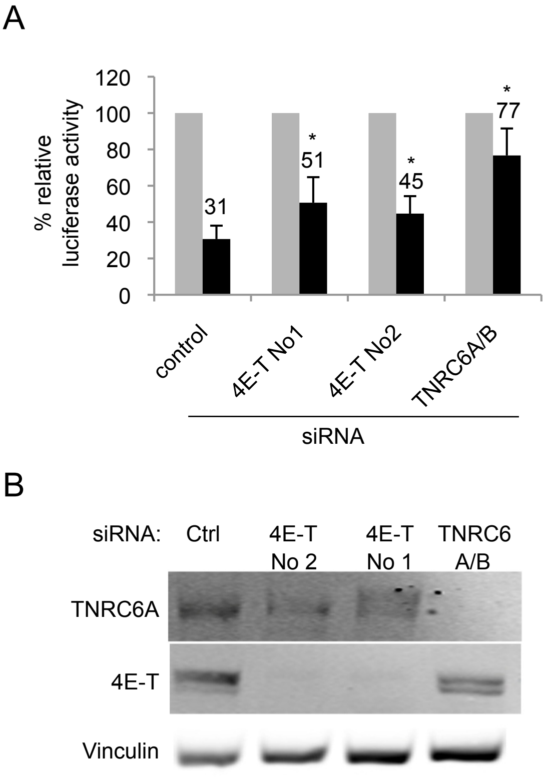

Suppl. Fig. 9

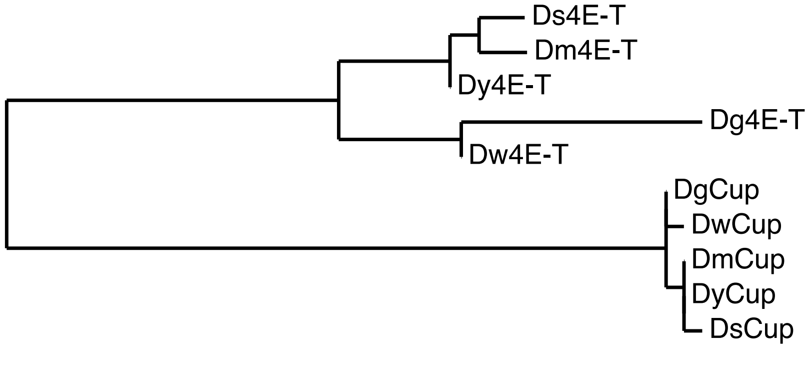

Suppl. Fig. 10

Supplementary Table 1

Primer	Sequence, 5’-3’	Purpose
4E-T-F0	GCGTTAGGTACCATGGATAGGAGAAGTATGGGT<br>KpnI	Forward primer for PCR and cloning h4E-T ORF in EGFP-C1 vector
4E-T-C1	CTCAAGCCCGGGTCACTGTCGGTATTCCAATTC<br>XmaI	Reverse primer for PCR and cloning h4E-T ORF in EGFP-C1 vector
Cup-F0	GCGTTACTCGAGTTATGCAAATGGCCGAAGCTGAG<br>XhoI	Forward primer for PCR and cloning Cup ORF in EGFP-C1 and EGFP-N1 vectors
Cup-REV-C1	CTCAAGCCCGGGTTAATGAAACTCATCCCGCT<br>XmaI	Reverse primer for PCR and cloning Cup ORF in EGFP-C1 vector
Cup-REV-N1	TGACCTCCCGGGTTAATGAAACTCATCCCCGCT<br>XmaI	Reverse primer for PCR and cloning Cup ORF in EGFP-N1 vector
Dm4E-T-FO	GCGTTAGGTACCATGGATACATCAAAGATTAGT<br>XhoI	Forward primer for PCR and cloning DmCg32016 ORF in EGFP-C1 and EGFP-N1 vectors
Dm4E-T-REV-C1	CTCAAGCCCGGGTTAGTTATGCACGACGCCTGA<br>XmaI	Reverse primer for PCR and cloning DmCg32016 ORF in EGFP-C1 vector
Dm4E-T-REV-N1	TGACCTCCCGGGCGTTATGCACGACGCCTGAGGA<br>XmaI	Reverse primer for PCR and cloning DmCg32016 ORF in EGFP-N1 vector
4E-T-Rev-C1_972	CTCAAGCCCGGGTTAGGGCATGGAGGGCAGGGG<br>XmaI	Reverse primer for PCR and cloning h4E-T_stop_972 ORF in EGFP-C1 vector
4E-T-Rev-C1_929	CTCAAGCCCGGGTTAGGGCACGTTCTGAGGGGT<br>XmaI	Reverse primer for PCR and cloning h4E-T_stop_929 ORF in EGFP-C1 vector

4E-T-Rev-C1_ 694	CTCAAG<u>CCCGGG</u>TTAAGGAGAAAGCATGCTTGT<br>XmaI	Reverse primer for PCR and cloning h4E-T_stop_694 ORF in EGFP-C1 vector
4E-T-Rev-C1_716	CTCAAG<u>CCCGGG</u>TTATGGCTCCTCCTTGCTTTT<br>XmaI	Reverse primer for PCR and cloning h4E-T_stop_716 ORF in EGFP-C1 vector
4E-T-Rev-C1_845	CTCAAG<u>CCCGGG</u>TTATGGAAGATGCTGTGGATG<br>XmaI	Reverse primer for PCR and cloning h4E-T_stop_845 ORF in EGFP-C1 vector
4E-TΔ694-716_D	AGCATGCTTTCTCCTGCATCTGGAAAAGCA	Reverse primer for PCR and cloning h4E-T_Δ694-716 ORF in EGFP-C1 vector
4E-TΔ694-716_B	TGCTTTTCCAGATGCAGGAGAAAGCATGCT	Reverse primer for PCR and cloning h4E-T_Δ694-716 ORF in EGFP-C1 vector
4E-TΔΔ_D	CTTGTCCCCCATGTCCCAAGTTTGCTCCAA	Reverse primer for PCR and cloning h4E-T_Δ694-716_Δ818-845 ORF in EGFP-C1 vector
4E-T ΔΔ_B	GTTTGGAGCAAACTTGGGACATGGGGGACACG	Reverse primer for PCR and cloning h4E-T_Δ694-716_Δ818-845 ORF in EGFP-C1 vector
4E-T-Rev-C1_Δ_808	CTCAAG<u>CCCGGG</u>TTAAGGGCGGAGAAAAGGTGT<br>XmaI	Reverse primer for PCR and cloning h4E-T_Δ694-716_stop_808 ORF in EGFP-C1 vector
4E-T-Start_695	GCGTTA<u>GGTACC</u>ATGTCCTTTACCCCTACC<br>KpnI	Forward primer for PCR and cloning h4E-T_start_695 ORF in EGFP-C1 vector
4E-T-Start_181	GCGTTA<u>GGTACC</u>ATGTTGAGAGACAGAGACCGA<br>KpnI	Forward primer for PCR and cloning h4E-

		T_start_181 ORF in EGFP-C1 vector
4E-T-Start_277	GCGTTAGGTACCATGTGGAGGTCATCCTTGCA<br>KpnI	Forward primer for PCR and cloning h4E-T_start_277 ORF in EGFP-C1 vector
4E-T-Start_354	GCGTTAGGTACCATGCTTGGGTCAACACCACAT<br>KpnI	Forward primer for PCR and cloning h4E-T_start_354 ORF in EGFP-C1 vector
4E-T-Start_551	GCGTTAGGTACCATGTTGGAGCCTACAACATCT<br>KpnI	Forward primer for PCR and cloning h4E-T_start_551 ORF in EGFP-C1 vector
4E-T-Start_601	GCGTTAGGTACCATGAGCCCCATCACAGCC<br>KpnI	Forward primer for PCR and cloning h4E-T_start_601 ORF in EGFP-C1 vector
4E-T-Start_677	GCGTTAGGTACCATGGTGACCAAGTCACCAGCA<br>KpnI	Forward primer for PCR and cloning h4E-T_start_677 ORF in EGFP-C1 vector
4E-T-Y30A-For	CAAATGCCCCCATCGCGCTACAAAAGAAGAACTC<br>Coding for Alanine	Forward primer for site-directed mutagenesis TAT89-91GCT in h4E-T-EGFP-C1
4E-T-Y30A-Rev	GAGTTCTTCTTTTGTAGCGCGATGGGGGCATTTG<br>Coding for Alanine	Reverse primer for site-directed mutagenesis TAT89-91GCT in h4E-T-EGFP-C1
4E-T-AAA-For	CAAATGCCCCCATCGCGCTACAAAAGAAGAAGCCGCGGATAT AAAAGAAC<br>Coding for Alanines	Forward primer for site-directed mutagenesis TAT89-91GCT, CTCTTG103-108GCCGCG in h4E-T-EGFP-C1
4E-T-AAA-Rev	GAGTTCTTTTATATCCGCGGCTTCTTCTTTTGTAGCGCGATG	Reverse primer for site-

	GGGGCATT<br>Coding for Alanines	directed mutagenesis TAT89-91GCT, CTCTTG103-108GCCGCG in h4E-T-EGFP-C1
4E-T_NLS_For	AAGGACAAGCGTTTCAACAGCGAGTTTGGAGATAGT<br>Coding for Asparagine and Serine	Forward primer for site-directed mutagenesis AGGAGA584-589AACAGC in h4E-T-EGFP-C1
4E-T_NLS_Rev	ACTATCTCCAAACTCGCTGTTGAAACGCTTGTCCTT<br>Coding for Asparagine and Serine	Reverse primer for site-directed mutagenesis AGGAGA584-589AACAGC in h4E-T-EGFP-C1
4E-TΔCHD_A	GAGAAGCTTCTATGGATAGGAGAAGTATG<br>HindIII	Forward primer for PCR and cloning h4E-T_ΔCHD ORF in EGFP-C1 vector
4E-TΔCHD_B	CTTCTTCTAGTATCTTCTCACCAAAGACACG	Reverse primer for PCR and cloning h4E-T_ΔCHD ORF in EGFP-C1 vector
4E-TΔCHD_C	GAGGGATCCTCACTGTCGGTATTCC<br>BamHI	Forward primer for PCR and cloning h4E-T_ΔCHD ORF in EGFP-C1 vector
4E-TΔCHD_D	CGTGTCTTTGGTGAGAAGATACTAGAAGAAG	Reverse primer for PCR and cloning h4E-T_ΔCHD ORF in EGFP-C1 vector
4E-T-HA_For	GCGAAGTCTAGAATGGATAGGAGAAGTATGGGT<br>XbaI	Forward primer for PCR and cloning h4E-T in EGFP-C1_HA_ΔGFP or EGFP-C1_NHA_ΔGFP vectors
4E-T_HA_Rev	GTCAAGGCGGCCGCTCACTGTCGGTATTCCAATTC<br>NotI	Reverse primer for PCR and cloning h4E-T in EGFP-C1_HA_ΔGFP or EGFP-C1_NHA_ΔGFP vectors
4E-T-HA-NheI	GCGTTAGCTAGCGATGGCCTACCCATATGATGTT<br>NheI	Forward primer for PCR and cloning HA-4E-T_NHA in EGFP-C1-ΔGFP vector
4E-T-NHA-NheI	GCGTTAGCTAGCGATGGACGCACAAACACGACGA	Forward primer for PCR

	NheI	and cloning NHA-4E-T_NHA in EGFP-C1-ΔGFP vector
GFP-HA_For	GCGAAGTCTAGAATGGTGAGCAAGGGCGAG<br>XbaI	Forward primer for site-directed mutagenesis TAT89-91GCT in h4E-T-EGFP-C1
GFP_HA_Rev	CTCAAGCCCGGGTTACTTGTACAGCTCGTCCAT<br>XmaI	Reverse primer for PCR and cloning GFP in EGFP-C1_HA_ΔGFP or EGFP-C1_NHA_ΔGFP vectors
BoxB_For	AGTAATTCTAGATAAGTCCAACTACTAAACTG<br>XbaI	Reverse primer for site-directed mutagenesis TAT89-91GCT in h4E-T-EGFP-C1
BoxB-Rev	AGGGAAGCGGCCGCCTCGAGATAATATCCTC<br>NotI	Reverse primer for site-directed mutagenesis TAT89-91GCT in h4E-T-EGFP-C1
qFluc_For	TCGCCAGTCAAGTAACAAC	Forward primer for RT-qPCR of Firefly luciferase mRNA
qFluc_Rev	ACTTCGTCCACCAACACA	Reverse primer for RT-qPCR of Firefly luciferase mRNA
qRluc_For	TCCGCAACTACAACGCCTAC	Forward primer for RT-qPCR of Renilla luciferase mRNA
qRluc_Rev	GGAACTTCTTAGCTCCCTCGAC	Reverse primer for RT-qPCR of Renilla luciferase mRNA
qGAPDH_For	TGCACCACCACCTGCTTAGC	Forward primer for RT-qPCR of GAPDH mRNA
qGAPDH_Rev	GGCATGGACTGTGGTCATGAG	Reverse primer for RT-qPCR of GAPDH mRNA
